# Computation of the Analytic Center of the Solution Set of the Linear Matrix Inequality Arising in Continuous- and Discrete-Time Passivity Analysis

**DOI:** 10.1007/s10013-020-00427-x

**Published:** 2020-07-23

**Authors:** Daniel Bankmann, Volker Mehrmann, Yurii Nesterov, Paul Van Dooren

**Affiliations:** 1 Institut für Mathematik MA 4-5, TU Berlin, Str. des 17. Juni 136, D-10623 Berlin, Germany; 2 Department of Mathematical Engineering, Université catholique de Louvain, Louvain-La-Neuve, Belgium

**Keywords:** Linear matrix inequality, Analytic center, Passivity, Robustness, Positive real system, Algebraic Riccati equation, 93D09, 93C05, 49M15, 37J25

## Abstract

In this paper formulas are derived for the analytic center of the solution set of linear matrix inequalities (LMIs) defining passive transfer functions. The algebraic Riccati equations that are usually associated with such systems are related to boundary points of the convex set defined by the solution set of the LMI. It is shown that the analytic center is described by closely related matrix equations, and their properties are analyzed for continuous- and discrete-time systems. Numerical methods are derived to solve these equations via steepest descent and Newton methods. It is also shown that the analytic center has nice robustness properties when it is used to represent passive systems. The results are illustrated by numerical examples.

## Introduction

We consider realizations of linear dynamical systems that are denoted as *positive real or passive* and their associated transfer functions. In particular, we study positive transfer functions which play a fundamental role in systems and control theory: they represent e.g., spectral density functions of stochastic processes, show up in spectral factorizations, are the Hermitian part of a positive real transfer function, characterize port-Hamiltonian systems, and are also related to algebraic Riccati equations.

Positive transfer functions form a convex set, and this property has lead to the extensive use of convex optimization techniques in this area (especially for so-called linear matrix inequalities [5]). In order to optimize a certain scalar function *f*(*X*) over a convex set, one often defines a barrier function *b*(*X*) that becomes infinite near the boundary of the set, and then finds the minimum of *c* ⋅ *f*(*X*) + *b*(*X*), *c* ≽ 0, as $c \rightarrow +\infty $. These minima (which are functions of the parameter *c*) are called the points of the *central path*. The starting point of this path (*c* = 0) is called the *analytic center* of the set. Notice that the analytic center depends as well on the barrier function as on the corresponding convex set.

In this paper we present an explicit set of equations that define the analytic center of the solution set of the linear matrix inequality defining a passive transfer function. We also show how these equations relate to the algebraic Riccati equations that typically arise in the spectral factorization of transfer functions. We discuss transfer functions both on the imaginary axis (i.e., the continuous-time case), as well as on the unit circle (i.e., the discrete-time case). In the continuous-time setting the transfer function arises from the *Laplace transform* of the system

1 $$  \begin{array}{rcl} \dot x & = & Ax + B u,~~x(0)=0,\\ y&=& Cx+Du, \end{array} $$

where $u:\mathbb R\to \mathbb {C}^{m}$, $x:\mathbb R\to \mathbb {C}^{n}$, and $y:\mathbb R\to \mathbb {C}^{m}$ are vector-valued functions denoting, respectively, the *input*, *state*, and *output* of the system. Denoting real and complex *n*-vectors (*n* × *m* matrices) by $\mathbb R^{n}$, $\mathbb C^{n}$ ($\mathbb R^{n \times m}$, $\mathbb {C}^{n \times m}$), respectively, the coefficient matrices satisfy $A\in \mathbb {C}^{n \times n}$, $B\in \mathbb {C}^{n \times m}$, $C\in \mathbb {C}^{m \times n}$, and $D\in \mathbb {C}^{m \times m}$.

In the discrete-time setting the transfer function arises from the *z-transform* applied to the system

$$ \begin{array}{@{}rcl@{}} x_{k+1} & = & Ax_{k} + B u_{k},~~x_{0}=0,\\ y_{k}&=& Cx_{k}+Du_{k}, \end{array} $$

with state, input, and output sequences {*x*_*k*_}, {*u*_*k*_}, {*y*_*k*_}. In both cases, we usually denote these systems by four-tuples of matrices ${\mathscr{M}}:=\{A,B,C,D\}$ and the associated transfer functions by

2 $$  \mathcal T_{c}(s):=D+C(sI_{n}-A)^{-1}B, \qquad \mathcal T_{d}(z):=D+C(z I_{n}-A)^{-1}B, $$

respectively.

We restrict ourselves to systems which are *minimal*, i.e., the pair (*A*,*B*) is *controllable* (for all $\lambda \in \mathbb C$, rank[*λI* − *AB*] = *n*), and the pair (*A*,*C*) is *reconstructable* (i.e., (*A*^*H*^,*C*^*H*^) is controllable). Here, the conjugate transpose (transpose) of a vector or matrix *V* is denoted by *V*^*H*^ (*V*^*T*^) and the identity matrix is denoted by *I*_*n*_ or *I* if the dimension is clear. We furthermore require that input and output port dimensions are equal to *m* and assume that rank*B* = rank*C* = *m*.

*Passive* systems and their relationships with *positive-real transfer functions* are well studied, starting with the works [13, 18, 21–24] and the topic has recently received a revival in the work on *port-Hamiltonian (pH) systems*, [15, 19, 20]. For a summary of the relationships see [2, 21], where also the characterization of passivity via the solution set of an associated *linear matrix inequality (LMI)* is highlighted.

The paper is organized as follows. After some preliminaries in Section 2, in Section 3 we study the analytic centers of the solution sets of LMIs associated with the continuous- and discrete-time case. In Section 4 we discuss numerical methods to compute the analytic centers using steepest descent as well as Newton methods and show that the analytic centers can be computed efficiently. In Section 5 lower bounds for the distance to non-passivity (the passivity radius) are derived using smallest eigenvalues of the Hermitian matrices associated with the linear matrix inequalities evaluated at the analytic center. The results are illustrated with some simple examples where the analytic center can be calculated analytically. In Appendix A we derive formulas for the computation of the gradients and the Hessian of the functions that we optimize and in Appendix B we clarify some of the differences that arise between the continuous- and the discrete-time case.

## Preliminaries

Throughout this article we will use the following notation. We denote the set of Hermitian matrices in $\mathbb {C}^{n \times n}$ by $\mathbb {H}_{n}$. Positive definiteness (semidefiniteness) of $A\in \mathbb {H}_{n}$ is denoted by *A* ≻ 0 (*A* ≽ 0). For a positive semi-definite matrix *A*, $\lambda _{\min \limits }(A)$ and $\lambda _{\max \limits }(A)$ denote, respectively, the smallest and largest eigenvalues of *A*. The real and imaginary parts of a complex matrix *Z* are written as *R*(*Z*) and *I*(*Z*), respectively, and *ı* is the imaginary unit. We consider functions over $\mathbb {H}_{n}$, which is a vector space if considered as a *real* subspace of $\mathbb {R}^{n\times n} + \imath \mathbb {R}^{n\times n}$. We will identify $\mathbb {C}^{m\times n}$ with $\mathbb {R}^{m\times n}+\imath \mathbb {R}^{m\times n}$, but we note that this has implications when one is carrying out differentiations, see Appendix A. The *Frobenius scalar product* for matrices $X, Y \in \mathbb {R}^{n\times n}+\imath \mathbb {R}^{n\times n}$ is given by

$$ \langle X,Y\rangle_{\mathbb{R}}:= \Re(\text{tr}(Y^{\mathsf{H}}X)) = \text{tr}({Y_{r}^{T}}X_{r}+{Y_{i}^{T}}X_{i}), $$

where we have partitioned *X*, *Y* as *X* = *X*_*r*_ + *ıX*_*i*_, *Y* = *Y*_*r*_ + *ıY*_*i*_ with real and imaginary parts in $\mathbb {R}^{n\times n}$. As we are mainly concerned with this scalar product, we will drop the subscript $\mathbb {R}$. We will make frequent use of the following properties of this inner product given by

$$ \langle X,Y\rangle = \langle Y,X\rangle, \quad \|X\|_{F}=\langle X,X\rangle^{\frac{1}{2}}, \quad \langle X,YZ\rangle = \langle Y^{\mathsf{H}}X,Z\rangle = \langle XZ^{\mathsf{H}},Y\rangle. $$

The concepts of *positive-realness* and *passivity* are well studied. In the following subsections we briefly recall some important properties following [10, 21], where we repeat a few observations from [2]. See also [21] for a more detailed survey.

### Positive-realness and Passivity, Continuous-time

Consider a continuous-time system ${\mathscr{M}}$ as in (1) and the transfer function $\mathcal T_{c}$ as in (2). The transfer function $\mathcal T_{c}(s)$ is called *positive real* if the matrix-valued rational function

$$ {\Phi}_{c}(s):= \mathcal T_{c}^{\mathsf{H}}(-s) + \mathcal T_{c}(s) $$

is positive semidefinite for *s* on the imaginary axis, i.e., Φ_*c*_(*ıω*) ≽ 0 for all $\omega \in \mathbb {R}$ and it is called *strictly positive real* if Φ_*c*_(*ıω*) ≻ 0 for all $\ \omega \in \mathbb {R}$.

We associate with Φ_*c*_ a system pencil
3where here and in the following *R* is an abbreviation for *R* := *D* + *D*^*H*^. Also, (3) has a Schur complement which is the transfer function Φ_*c*_(*s*) and, under the condition of minimality, the finite generalized eigenvalues of *S*_*c*_(*s*) are the finite zeros of Φ_*c*_(*s*).

For $X \in \mathbb {H}_{n}$ we introduce the matrix function

4 $$  W_{c}(X) := \left[ \begin{array}{cc} -XA - A^{\mathsf{H}}X &~C^{\mathsf{H}} - XB \\ C- B^{\mathsf{H}}X &~R \end{array} \right]. $$

If $\mathcal T_{c}(s)$ is positive real, then the linear matrix inequality (LMI)

5 $$  W_{c}(X) \succeq 0 $$

has a solution $X\in \mathbb {H}_{n}$ and we have the sets

6a $$ \begin{array}{@{}rcl@{}} \mathbb{X}_{c}^{\succ}&:=&\{X\in \mathbb{H}_{n} ~|~ W_{c}(X) \succeq 0,~X \succ 0\}, \end{array} $$

6b $$ \begin{array}{@{}rcl@{}} \mathbb{X}_{c}^{\succ\!\!\succ} &:=&\{X\in \mathbb{H}_{n}~|~W_{c}(X) \succ 0,~X \succ 0\}. \end{array} $$

An important subset of $\mathbb {X}_{c}^{\succ }$ are those solutions to (5) for which the rank *r* of *W*_*c*_(*X*) is minimal (i.e., for which *r* = rankΦ_*c*_(*s*)). If *R* is invertible, then the minimum rank solutions in $\mathbb {X}^{\succ }_{c}$ are those for which rank*W*_*c*_(*X*) = rank(*R*) = *m*, which in turn is the case if and only if the Schur complement of *R* in *W*_*c*_(*X*) is zero. This Schur complement is associated with the continuous-time *algebraic Riccati equation (ARE)*

7 $$ \mathsf{Ricc}_{c}(X) := -XA-A^{\mathsf{H}}X -(C^{\mathsf{H}}-XB)R^{-1}(C-B^{\mathsf{H}}X)=0.  $$

Solutions *X* to (7) produce a spectral factorization of Φ_*c*_(*s*), and each solution corresponds to a *Lagrangian invariant subspace* spanned by the columns of $U_{c}:=\left [\begin {array}{cc} I_{n} &~-X^{\mathsf {T}} \end {array} \right ]^{\mathsf {T}}$ that remains invariant under the action of the *Hamiltonian matrix*

8 $$ \mathcal{H}_{c}:=\left[\begin{array}{cc} A-B R^{-1} C & - B R^{-1} B^{\mathsf{H}} \\ C^{\mathsf{H}} R^{-1} C & -(A-B R^{-1} C)^{\mathsf{H}} \end{array}\right], $$

i.e., *U*_*c*_ satisfies ${\mathscr{H}}_{c}U_{c}=U_{c} A_{F_{c}}$ for a *closed loop matrix* $A_{F_{c}}=A-BF_{c}$ with *F*_*c*_ := *R*^− 1^(*C* − *B*^*H*^*X*) (see e.g., [8]). Each solution *X* of (7) can also be associated with an *extended Lagrangian invariant subspace* for the pencil *S*_*c*_(*s*) (see [4]), spanned by the columns of $\widehat {U}_{c}:=\left [\begin {array}{ccc} -X^{\mathsf {T}} & I_{n} & -F_{c}^{\mathsf {T}} \end {array}\right ]^{\mathsf {T}}$. In particular, $\widehat {U}_{c}$ satisfies

$$ \left[ \begin{array}{ccc} 0 & A & B \\ A^{\mathsf{H}} & 0 & C^{\mathsf{H}} \\ B^{\mathsf{H}} & C & R \end{array} \right] \widehat{U}_{c} =\left[ \begin{array}{ccc} 0 & I_{n} & 0\\ -I_{n} & 0 & 0\\ 0 & 0 & 0 \end{array} \right] \widehat{U}_{c} A_{F_{c}}. $$

The sets $\mathbb {X}^{\succ }_{c}$, $\mathbb {X}^{\succ \!\!\succ }_{c}$ are related to the concepts of *passivity and strict passivity* see [21]. If for the system ${\mathscr{M}}:=\{A,B,C,D\}$ of (3) the LMI (5) has a solution $X\in \mathbb {X}^{\succ }_{c}$ then ${\mathscr{M}}$ is *(Lyapunov) stable* (i.e., all eigenvalues are in the closed left half plane with any eigenvalues occurring on the imaginary axis being semisimple), and *passive*, and if there exists a solution $ X\in \mathbb {X}^{\succ \!\!\succ }_{c}$ then ${\mathscr{M}}$ is *asymptotically stable* (i.e., all eigenvalues are in the open left half plane) and *strictly passive*. Furthermore, if ${\mathscr{M}}$ is passive, then there exist maximal and minimal solutions *X*_−_≼ *X*_+_ of (5) in $\mathbb {X}^{\succ }_{c}$ such that all solutions *X* of *W*_*c*_(*X*) ≽ 0 satisfy

$$ 0 \prec X_{-} \preceq X \preceq X_{+}, $$

which implies that $\mathbb {X}^{\succ }_{c}$ is bounded. For more details on the different concepts discussed in this section, see the extended preprint version of [2].

### Positive-realness and Passivity, Discrete-time

For each of the results of the previous subsection there are discrete-time versions which we briefly recall in this section, see [12, 18]. Note, that these results can be obtained by applying a bilinear transform (see Appendix B) to the continuous-time counterparts.

The transfer function $\mathcal T_{d}(z)$ in (2) is called *positive real* if the matrix-valued rational function

$$ {\Phi}_{d}(z):= \mathcal T_{d}^{\mathsf{H}}(z^{-1}) + \mathcal T_{d}(z) $$

satisfies $ {\Phi }_{d}(e^{\imath \omega }) = {\Phi }_{d}^{\mathsf {H}}(e^{\imath \omega }) \succeq 0 $ for 0 ≤ *ω* ≤ 2*π*, and it is called *strictly positive real* if Φ_*d*_(*e*^*ıω*^) ≻ 0 for 0 ≤ *ω* ≤ 2*π*.

We consider an associated the matrix function

$$ W_{d}(X) = \left[ \begin{array}{cc} X-A^{\mathsf{H}}X A &~C^{\mathsf{H}}-A^{\mathsf{H}}X B \\ C-B^{\mathsf{H}}X A &~R-B^{\mathsf{H}}X B \end{array} \right], $$

where again *R* = *D* + *D*^*H*^, the sets

9a $$ \begin{array}{@{}rcl@{}} \mathbb{X}^{\succ}_{d} &:=& \{X\in \mathbb{H}_{n} ~|~ W_{d}(X) \succeq 0,~X \succ 0\}, \end{array} $$

9b $$ \begin{array}{@{}rcl@{}} \mathbb{X}^{\succ\!\!\succ}_{d} &:=& \{X\in \mathbb{H}_{n} ~|~ W_{d}(X) \succ 0,~X \succ 0\}, \end{array} $$

and the system pencil
 whose Schur complement is Φ_*d*_(*z*).

If the system is positive real then, see [21], there exists $X\in \mathbb {H}_{n}$ such that *W*_*d*_(*X*) ≽ 0. If *W*_*d*_(*X*) ≽ 0, a transfer function $\mathcal {T}_{d}(z):=C(zI_{n}-A)^{-1}B+D$ is called *passive* and if *W*_*d*_(*X*) ≻ 0 it is said to be *strictly passive*. We again have an associated discrete-time Riccati equation defined as

10 $$ \mathsf{Ricc}_{d}(X) :=-A^{\mathsf{H}}XA+X-(C^{\mathsf{H}}-A^{\mathsf{H}} X B) (R-B^{\mathsf{H}} X B)^{-1}(C-B^{\mathsf{H}}X A) =0  $$

from which one directly obtains a spectral factorization of Φ_*d*_(*z*). The solutions of the discrete-time Riccati equation can be obtained by computing a Lagrangian invariant subspace spanned by the columns of $U_{d}:=\left [\begin {array}{cc} I_{n} &~-X^{\mathsf {T}} \end {array} \right ]^{\mathsf {T}}$ of the *symplectic matrix*

$$ \mathcal{S}_{d} := \left[\begin{array}{cc} I &~BR^{-1}B^{\mathsf{H}} \\ 0 &~A^{\mathsf{H}}-C^{\mathsf{H}}R^{-1}B^{\mathsf{H}} \end{array} \right]^{-1} \left[\begin{array}{cc} A-BR^{-1}C &~0 \\ C^{\mathsf{H}}R^{-1}C &~I \end{array} \right], $$

satisfying $\mathcal {S}_{d} U_{d}=U_{d} A_{F_{d}}$, where $A_{F_{d}} := A-BF_{d}$ with *F*_*d*_ := (*R* − *B*^*H*^*XB*)^− 1^(*C* − *B*^*H*^*XA*).

Each solution *X* of (10) can also be associated with an *extended Lagrangian invariant subspace* for the pencil *S*_*d*_(*z*) (see [4]), spanned by the columns of $\widehat {U}_{d}:=\left [\begin {array}{ccc} -X^{\mathsf {T}}&~I_{n} &~-F_{d}^{\mathsf {T}} \end {array}\right ]^{\mathsf {T}}$. In particular, $\widehat {U}_{d}$ satisfies

$$ \left[ \begin{array}{ccc} 0 & A & B \\ I_{n} & 0 & C^{\mathsf{H}} \\ 0 & C & R \end{array} \right] \widehat{U}_{d} =\left[ \begin{array}{ccc} 0 & I_{n} & 0\\ A^{\mathsf{H}} & 0 & 0\\ B^{\mathsf{H}} & 0 & 0 \end{array} \right] \widehat{U}_{d} A_{F_{d}}. $$

Again, if the system is passive, then there exist maximal and minimal solutions *X*_−_≼ *X*_+_ in $\mathbb {X}^{\succ }_{d}$, such that all solutions *X* of *W*_*d*_(*X*) ≽ 0 satisfy

$$ 0 \prec X_{-} \preceq X \preceq X_{+}, $$

which implies that $\mathbb {X}^{\succ }_{d}$ is bounded.

## The Analytic Center

If the sets $\mathbb {X}^{\succ \!\!\succ }_{c}$, $\mathbb {X}^{\succ \!\!\succ }_{d}$ in (2.1), respectively (2.2), are non-empty, then we can define their respective analytic center. Following the discussion in [10], we first consider the continuous-time case, the discrete-time case is derived in an analogous way. We choose a scalar barrier function

$$ b(X) := -\ln \det W_{c}(X), \quad X\in \mathbb{H}_{n} $$

which is bounded from below but becomes infinitely large when *W*_*c*_(*X*) becomes singular. We define the analytic center of the domain $\mathbb {X}^{\succ \!\!\succ }_{c}$ as the minimizer of this barrier function.

### The Continuous-time Case

Since $\mathbb {X}^{\succ \!\!\succ }_{c}$ is non-empty, *R* is invertible and the Riccati equation *Ricc*_*c*_(*X*) = 0 in (7) is well defined, Their solutions *X*_+_ and *X*_−_ are both on the boundary of $\mathbb {X}^{\succ }_{c}$, and hence are not in $\mathbb {X}^{\succ \!\!\succ }_{c}$. Since we assume that $\mathbb {X}^{\succ \!\!\succ }_{c}$ is non-empty, the analytic center is well defined, see, e.g., Section 4.2 in [17].

To characterize the analytic center, we need to consider the variation of the *gradientb*_*X*_ of the barrier function *b* at point *X* along a direction ${\Delta }_{X}\in \mathbb {H}_{n}$. As explained in Appendix A, this is equal to

11 $$ -\langle W_{c}(X)^{-1},{\Delta} W_{c}(X)[{\Delta}_{X}]\rangle, $$

where *b*_*X*_ = −*W*_*c*_(*X*)^− 1^ and Δ*W*_*c*_(*X*)[Δ_*X*_] is the incremental step in the direction Δ_*X*_. It appears that *X* is an extremal point of the barrier function if and only if

$$ -\langle W_{c}(X)^{-1}, {\Delta} W_{c}(X)[{\Delta}_{X}]\rangle = 0\quad \text{ for all }{\Delta}_{X}\in\mathbb{H}_{n}. $$

The increment of *W*_*c*_(*X*) corresponding to an incremental direction ${\Delta }_{X}\in \mathbb {H}_{n}$ of *X* is given by

$$ {\Delta} W_{c}(X)[{\Delta}_{X}] = -\left[ \begin{array}{cc} A^{\mathsf{H}}{\Delta}_{X}+{\Delta}_{X} A &~{\Delta}_{X} B \\ B^{\mathsf{H}}{\Delta}_{X} &~0 \end{array} \right]. $$

The equation for the extremal point then becomes

12 $$ \left\langle W_{c}(X)^{-1},\left[\begin{array}{cc} A^{\mathsf{H}}{\Delta}_{X} + {\Delta}_{X} A &~{\Delta}_{X} B \\ B^{\mathsf{H}}{\Delta}_{X} &~ 0 \end{array} \right]\right\rangle = 0\quad \text{for all }{\Delta}_{X}\in\mathbb{H}_{n}. $$

Defining

$$ F_{c} := R^{-1}(C-B^{\mathsf{H}}X), \quad P_{c} := -A^{\mathsf{H}}X-XA-F_{c}^{\mathsf{H}}RF_{c}, $$

then

$$ W_{c}(X)= \left[ \begin{array}{cc} I & F_{c}^{\mathsf{H}} \\ 0 & I \end{array} \right] \left[ \begin{array}{cc} P_{c} & 0 \\ 0 & R \end{array} \right] \left[ \begin{array}{cc} I & 0 \\ F_{c} & I \end{array} \right]. $$

For a point $X\in \mathbb {X}^{\succ \!\!\succ }_{c}$ it is obvious that we also have *P*_*c*_ = *Ricc*_*c*_(*X*) ≻ 0, and hence (12) is equivalent to

$$ \left\langle \left[ \begin{array}{cc} P_{c}^{-1} & 0 \\ 0 & R^{-1} \end{array} \right], \left[ \begin{array}{cc} I & -F_{c}^{\mathsf{H}} \\ 0 & I \end{array} \right] \left[ \begin{array}{cc} A^{\mathsf{H}}{\Delta}_{X}+{\Delta}_{X} A & {\Delta}_{X} B \\ B^{\mathsf{H}}{\Delta}_{X} & 0 \end{array} \right] \left[ \begin{array}{cc} I & 0 \\ -F_{c} & I \end{array} \right]\right\rangle = 0, $$

or

$$ \langle P_{c}^{-1}, A^{\mathsf{H}}{\Delta}_{X}+{\Delta}_{X} A-F_{c}^{\mathsf{H}}B^{\mathsf{H}}{\Delta}_{X}-{\Delta}_{X} BF_{c} \rangle =0 \quad \text{for all }{\Delta}_{X}\in\mathbb{H}_{n}, $$

which is equivalent to

$$ \langle P_{c}^{-1}A^{\mathsf{H}}_{F_{c}} + A_{F_{c}} P_{c}^{-1}, {\Delta}_{X}\rangle =0 \quad \text{for all }{\Delta}_{X}\in\mathbb{H}_{n}, $$

where we have set $A_{F_{c}} = A-BF_{c}$. This then implies

13 $$  P_{c}^{-1}A_{F_{c}}^{\mathsf{H}} +A_{F_{c}} P_{c}^{-1} =0. $$

We emphasize that *P*_*c*_ is nothing but the Riccati operator *Ricc*_*c*_(*X*) defined in (7), and that $A_{F_{c}}$ is the corresponding closed loop matrix. For the classical Riccati solutions we have *P*_*c*_ = *Ricc*_*c*_(*X*) = 0 and the corresponding closed loop matrix is well-known to have its eigenvalues equal to a subset of the eigenvalues of the corresponding Hamiltonian matrix (8).

Since *P*_*c*_ = *Ricc*_*c*_(*X*) ≻ 0, it follows that *P*_*c*_ has a Hermitian square root *T*_*c*_ satisfying $P_{c}={T_{c}^{2}}$. Transforming (13) with the invertible matrix *T*_*c*_, we obtain

$$ T_{c}^{-1}A_{F_{c}}^{\mathsf{H}}T_{c} + T_{c}A_{F_{c}}T_{c}^{-1}=0. $$

Hence, $\hat A_{F_{c}}:=T_{c}A_{F_{c}}T_{c}^{-1}$ is skew-Hermitian and has all its eigenvalues on the imaginary axis, and so does $A_{F_{c}}$. Therefore, the closed loop matrix $A_{F_{c}}$ of the analytic center has a spectrum that is also central.

It is important to also note that

$$ \det W_{c}(X) = \det \mathsf{Ricc}_{c}(X) \det R, $$

which implies that we are also finding a stationary point of $\det \mathsf {Ricc}_{c}(X)$, since $\det R$ is constant and non-zero.

Since the matrix *P*_*c*_ is positive definite and invertible, we can rewrite the equations defining the analytic center as

$$ \begin{array}{@{}rcl@{}} RF_{c} &=& C-B^{\mathsf{H}}X,\\ P_{c} &=& -A^{\mathsf{H}}X-X A-F_{c}^{\mathsf{H}}RF_{c}, \\ 0 &=& P_{c}(A-BF_{c})+(A^{\mathsf{H}}-F_{c}^{\mathsf{H}}B^{\mathsf{H}})P_{c}, \end{array} $$

where *X* = *X*^*H*^ and $P_{c}=P_{c}^{\mathsf {H}} \succ 0$. We can compute the analytic center by solving these three equations which actually form a cubic equation in *X*. Note that due to the convexity of the problem, the analytic center is the only solution of these equations where the conditions $X \in \mathbb {X}^{\succ \!\!\succ }_{c}$ and *P*_*c*_ ≻ 0 are both met.

Note that even though the eigenvalues of the closed loop matrix $A_{F_{c}}$ associated with the analytic center are all purely imaginary, the eigenvalues of the original system and the poles of the transfer function stay invariant under the state space transformation *T*_*c*_.

#### *Remark 1* (Interpretation of the analytic center)

For strictly positive real systems, the set of strictly positive LMI solutions $\mathbb {X}^{\succ \!\!\succ }_{c}$ contains infinitely many elements. Every solution $X\in \mathbb {X}^{\succ \!\!\succ }_{c}$ defines a port-Hamiltonian realization of which the analytic center is most robust in terms of conditioning, see [2] for more details.

### The Discrete-time Case

For discrete-time systems, the increment of *W*_*d*_(*X*) equals

$$ {\Delta} W_{d}(X)[{\Delta}_{X}] = - \left[ \begin{array}{cc} A^{\mathsf{H}}{\Delta}_{X}A-{\Delta}_{X} &~A^{\mathsf{H}}{\Delta}_{X}B \\ B^{\mathsf{H}}{\Delta}_{X}A &~B^{\mathsf{H}}{\Delta}_{X}B \end{array} \right] $$

for all ${\Delta }_{X}\in \mathbb {H}_{n}$. Defining *F*_*d*_ := (*R* − *B*^*H*^*XB*)^− 1^(*C* − *B*^*H*^*XA*), $A_{F_{d}} := A-BF_{d}$, and $P_{d} := X - A^{\mathsf {H}}XA -F_{d}^{\mathsf {H}}(R-B^{\mathsf {H}}XB)F_{d}$, then *W*_*d*_(*X*) factorizes as

$$ W_{d}(X)= \left[ \begin{array}{cc} I & F_{d}^{\mathsf{H}} \\ 0 & I \end{array} \right] \left[ \begin{array}{cc} P_{d} &~0 \\ 0 &~R-B^{\mathsf{H}}XB \end{array} \right] \left[ \begin{array}{cc} I & 0 \\ F_{d} & I \end{array} \right], $$

and the equation for the extremal point becomes

$$ \begin{array}{@{}rcl@{}} && \bigg\langle \left[ \begin{array}{cc} P_{d}^{-1} & 0 \\ 0 & (R-B^{\mathsf{H}}XB)^{-1} \end{array} \right],\\ && \qquad \left[ \begin{array}{cc} I & -F_{d}^{\mathsf{H}} \\ 0 & I \end{array} \right] \left[ \begin{array}{cc} A^{\mathsf{H}}{\Delta}_{X} A-{\Delta}_{X} &~A^{\mathsf{H}}{\Delta}_{X} B \\ B^{\mathsf{H}}{\Delta}_{X} A &~B^{\mathsf{H}}{\Delta}_{X} B \end{array} \right] \left[ \begin{array}{cc} I & 0 \\ -F_{d} & I \end{array} \right] \bigg \rangle =0 \quad \text{for all }{\Delta}_{X}\in\mathbb{H}_{n}, \end{array} $$

or

$$ \langle P_{d}^{-1}, A_{F_{d}}^{\mathsf{H}}{\Delta}_{X} A_{F_{d}}-{\Delta}_{X} \rangle + \langle (R-B^{\mathsf{H}}XB)^{-1}, B^{\mathsf{H}}{\Delta}_{X} B\rangle =0 \quad \text{for all }{\Delta}_{X}\in\mathbb{H}_{n}. $$

This is equivalent to

14 $$  A_{F_{d}}P_{d}^{-1}A_{F_{d}}^{\mathsf{H}} -P_{d}^{-1}+B(R-B^{\mathsf{H}}XB)^{-1}B^{\mathsf{H}} =0, $$

which can be seen as a discrete-time Lyapunov equation if *X* was fixed and independent of *P*_*d*_. Since (*A*,*B*) is controllable (by assumption), so is $(A_{F_{d}},B)$ and it follows then from (14) that the eigenvalues of $A_{F_{d}}$ are now strictly inside the unit circle. This is *clearly different from the continuous-time case*, where the spectrum of $A_{F_{c}}$ was on the boundary of the stability region. The equations defining the discrete-time analytic center then become

$$ \begin{array}{@{}rcl@{}} (R-B^{\mathsf{H}}X B)F_{d} &=& C-B^{\mathsf{H}}X A,\\ P_{d} &=& X-A^{\mathsf{H}}X A-F_{d}^{\mathsf{H}}(R-B^{\mathsf{H}}X B)F_{d}, \\ 0 &=& (A-BF_{d})P_{d}^{-1}(A^{\mathsf{H}}-F_{d}^{\mathsf{H}}B^{\mathsf{H}}) -P_{d}^{-1}+ B(R-B^{\mathsf{H}}X B)^{-1}B^{\mathsf{H}}. \end{array} $$

#### *Remark 2*

Note that the solution of the discrete-time problem does not coincide with with the one obtained via a bilinear transformation of the continuous-time problem, since this would yield a feedback *F*_*d*_ that puts all eigenvalues on the unit circle. The bilinear transformation does not preserve determinants, and therefore the solution of the minimization problem can be expected to be different (see also Appendix B).

## Numerical Computation of the Analytic Center

In this section we present methods for the numerical computation of the analytic center.

Suppose that we are at a point $X_{0} \in \mathbb {X}^{\succ \!\!\succ }_{c}~ (\mathbb {X}^{\succ \!\!\succ }_{d})$ and want to perform the next step using an increment Δ_*X*_. We discuss a steepest descent and a Newton method to obtain that increment.

### A Steepest Descent Method

In order to formulate an optimization scheme to compute the analytic center, we can use the gradient of the barrier function *b*(*X*) with respect to *X* in a point *X*_0_ to obtain a steepest descent method.

In the continuous-time case, we then need to take a step Δ_*X*_ for which 〈*b*(*X*_0_),Δ*W*_*c*_(*X*_0_)[Δ_*X*_]〉 is minimized, which is equivalent to

$$ {\Delta}_{X} := \underset{\langle{\Delta}_{X}, {\Delta}_{X}\rangle=1}{\arg\min} \left\langle {\Delta}_{X}, P_{c}^{-1}(X_{0})A_{F_{c}}(X_{0})^{\mathsf{H}}+A_{F_{c}}(X_{0})P_{c}^{-1}(X_{0})\right\rangle. $$

The minimum is obtained by choosing Δ_*X*_ proportional to the gradient

$$ P_{c}^{-1}(X_{0})A_{F_{c}}(X_{0})^{\mathsf{H}}+A_{F_{c}}(X_{0})P_{c}^{-1}(X_{0}). $$

The corresponding optimal stepsize *α* for the increment Δ_*X*_ can be obtained from the determinant of the incremented LMI *W*_*c*_(*X*_0_ + *α*Δ_*X*_) ≻ 0.

In the discrete-time case, we obtain the increment from

$$ {\Delta}_{X} := \underset{\langle{\Delta}_{X}, {\Delta}_{X}\rangle=1}{\arg\min} \left\langle{\Delta}_{X}, A_{F_{d}}(X_{0})P_{d}^{-1}(X_{0})A_{F_{d}}^{\mathsf{H}}(X_{0})-P_{d}^{-1}(X_{0})+B(R-B^{\mathsf{H}}X_{0}B)^{-1}B^{\mathsf{H}}\right\rangle. $$

The minimum is obtained by choosing Δ_*X*_ proportional to the gradient

$$ A_{F_{d}}(X_{0}) P_{d}^{-1}(X_{0})A_{F_{d}}^{\mathsf{H}}(X_{0})-P_{d}^{-1}(X_{0}) + B(R-B^{\mathsf{H}}X_{0}B)^{-1}B^{\mathsf{H}}, $$

and the stepsize *α* for the increment Δ_*X*_ can again be obtained from the determinant of the incremented LMI *W*_*d*_(*X*_0_ + *α*Δ_*X*_) ≻ 0.

#### *Remark 3*

The detailed explanation how to compute the stepsize *α* will be done later as a special case of the derivation of the Newton step, see Section 4.2. The idea is to find the second order Taylor expansion of the function $b(X_{0}+\alpha {\Delta }_{X})=-\ln \det W(X_{0}+\alpha {\Delta }_{X})$ and then to minimize this quadratic function in the scalar *α*. This is a one dimensional Newton step and will only yield an inexact line-search.

### Newton Method

For the computation of a Newton step Δ_*X*_ we also need the Hessian of the barrier function *b*. In order to simplify the derivation of the Hessian, we first reformulate the determinant of *W*(*X*_0_ + Δ_*X*_) into a more suitable form. We also point out that minimizing $-\ln \det W(X)$ is equivalent to maximizing $\det W(X)$.

#### The Continuous-time Case

In the continuous-time case, we have that

$$ W_{c} (X_{0}+{\Delta}_{X}) = \left[ \begin{array}{cc} Q_{0} & C_{0}^{\mathsf{H}}\\ C_{0} & R_{0} \end{array} \right] - \left[\begin{array}{c} {\Delta}_{X}\\ 0 \end{array} \right] \left[\begin{array}{cc} A &~B \end{array} \right] - \left[\begin{array}{c} A^{\mathsf{H}} \\ B^{\mathsf{H}} \end{array} \right] \left[\begin{array}{cc} {\Delta}_{X} &~0 \end{array} \right], $$

where

$$ \left[ \begin{array}{cc} Q_{0} & C_{0}^{\mathsf{H}}\\ C_{0} & R_{0} \end{array} \right] := W_{c} (X_{0}). $$

By taking Schur complements and applying congruence transformations, it follows that the product $\det W_{c} (X_{0}+{\Delta }_{X})(-1)^{n}$ is equal to
 where $A_{F_{c}}:=A-BR_{0}^{-1}C_{0}$ and $P_{0}:=Q_{0}-C_{0}^{\mathsf {H}}R_{0}^{-1}C_{0}$ are associated with the current point *X*_0_. Carrying out an additional congruence transformation with

$$ Z_{c} := \left[\begin{array}{cccc} P_{0}^{-\frac{1}{2}} & 0 & 0 & 0 \\ 0 & P_{0}^{\frac{1}{2}} & 0 & -\hat BR_{0}^{-1} \\ 0 & 0 & P_{0}^{-\frac{1}{2}}& 0 \\ 0 & 0 & 0 & R_{0}^{-\frac{1}{2}} \end{array}\right], $$

we obtain

15 $$  \left[\begin{array}{cccc} 0 & I_{n} & \hat {\Delta}_{X} & 0 \\ I_{n} & -\hat BR_{0}^{-1}\hat B^{\mathsf{H}} & \hat A_{F_{c}} & 0 \\ \hat {\Delta}_{X} & \hat A_{F_{c}}^{\mathsf{H}} & I_{n} & 0 \\ 0 & 0 & 0 & I_{m} \end{array}\right] := Z_{c} \left[\begin{array}{cccc} 0 & I_{n} & {\Delta}_{X} & 0 \\ I_{n} & 0 & A_{F_{c}} & B \\ {\Delta}_{X} & A_{F_{c}}^{\mathsf{H}} & P_{0} & 0 \\ 0 & B^{\mathsf{H}} & 0 & R_{0} \end{array}\right] Z_{c}^{\mathsf{H}}, $$

where $\hat B = P_{0}^{\frac {1}{2}} B$, $\hat A_{F_{c}}:=P_{0}^{\frac {1}{2}}A_{F_{c}}P_{0}^{-\frac {1}{2}}$, and $\hat {\Delta }_{X}=P_{0}^{-\frac {1}{2}}{\Delta }_{X}P_{0}^{-\frac {1}{2}}$. It is clear that the determinant of the congruence transformation *Z*_*c*_ is given by

16 $$  (\det Z_{c})^{2}= 1/(\det P_{0}\cdot \det R_{0})=1/\det W_{c}(X_{0}). $$

The above transformations finally lead to the following lemma.

##### **Lemma 1**

The change of variables

$$ \hat B = P_{0}^{\frac{1}{2}} B, \quad \hat A_{F_{c}}:=P_{0}^{\frac{1}{2}}A_{F_{c}}P_{0}^{-\frac{1}{2}}, \quad \hat {\Delta}_{X}=P_{0}^{-\frac{1}{2}}{\Delta}_{X}P_{0}^{-\frac{1}{2}}, \quad \hat X=P_{0}^{-\frac{1}{2}}XP_{0}^{-\frac{1}{2}} $$

yields the following determinant identity

17 $$ \begin{array}{@{}rcl@{}} \det W_{c}(X_{0}+{\Delta}_{X}) &= & \det \left[\begin{array}{ccc} 0 & I_{n} & \hat {\Delta}_{X} \\ I_{n} & -\hat BR_{0}^{-1}\hat B^{\mathsf{H}} & \hat A_{F_{c}}\\ \hat {\Delta}_{X} & \hat A_{F_{c}}^{\mathsf{H}} & I_{n} \end{array}\right] \end{array} $$

18 $$ \begin{array}{@{}rcl@{}} &= & \det \left[I_{n} - \hat {\Delta}_{X} \hat A_{F_{c}} - \hat A_{F_{c}}^{\mathsf{H}} \hat {\Delta}_{X} - \hat {\Delta}_{X}\hat BR_{0}^{-1}\hat B^{\mathsf{H}} \hat {\Delta}_{X} \right]. \end{array} $$

##### *Proof*

The determinant of the right hand side of (15) equals $\det W_{c}(X_{0}+{\Delta }_{X})$ because of (16). The equalities (17), (18) then easily follow. □

We thus have an equivalent minimization problem $\min \limits _{X\in \mathbb {H}_{n}} f(X)$ in the new “translated” variable $X=\hat {\Delta }_{X}$ corresponding to an initial point at the origin of the barrier function

$$ \begin{array}{@{}rcl@{}} f(X)&:=& - \ln \det (G(X)),\\ Q_{c}&:=&\hat BR_{0}^{-1}\hat B^{\mathsf{H}},\\ G(X)&:=& I_{n} - X \hat A_{F_{c}} - \hat A_{F_{c}}^{\mathsf{H}} X - XQ_{c}X. \end{array} $$

In the set of Hermitian matrices (over the reals), the gradient of *f*(*X*) then is given by

$$ f_{X}(X)[{\Delta}] = \langle- G(X)^{-1},-({\Delta} \hat A_{F_{c}} + \hat A_{F_{c}}^{\mathsf{H}} {\Delta} + {\Delta} Q_{c} X + X Q_{c} {\Delta})\rangle $$

and the Hessian is given by

$$ \begin{array}{@{}rcl@{}} f_{XX}(X)[{\Delta},{\Delta}] &=& \left\langle -G(X)^{-1}({\Delta} \hat A_{F_{c}} + \hat A_{F_{c}}^{\mathsf{H}} {\Delta} + {\Delta} Q_{c} X + X Q_{c}{\Delta})G(X)^{-1},\right .\\ && \quad \left. -({\Delta} \hat A_{F_{c}} + \hat A_{F_{c}}^{\mathsf{H}} {\Delta} + {\Delta} Q_{c} X + X Q_{c}{\Delta})\right\rangle \\ && + \langle -G(X)^{-1}, -2{\Delta} Q_{c} {\Delta} \rangle. \end{array} $$

A second order approximation of *f* (at *X* = 0) is given by

$$ \begin{array}{@{}rcl@{}} f({\Delta}) \approx T^{(2)}_{f}({\Delta}) &=& f(0) + f_{X}(0)[{\Delta}] + \frac{1}{2}f_{XX}(0)[{\Delta}, {\Delta}]\\ & = & \langle I_{n}, {\Delta} \hat A_{F_{c}} + \hat A_{F_{c}}^{\mathsf{H}} {\Delta} \rangle + \frac{1}{2} \langle {\Delta} \hat A_{F_{c}} + \hat A_{F_{c}}^{\mathsf{H}} {\Delta}, {\Delta} \hat A_{F_{c}} + \hat A_{F_{c}}^{\mathsf{H}} {\Delta} \rangle\\ &&+ \langle I_{n}, {\Delta} Q_{c} {\Delta} \rangle. \end{array} $$

Remember, that in order to minimize *f*(*X*) we want the gradient of *f* to be 0. Thus, for the Newton step we want to determine Δ = Δ^*H*^ such that $\frac {\partial T^{(2)}_{f}}{\partial {\Delta }}({\Delta })[Y]=0$ for all $Y\in \mathbb {H}_{n}$, i.e., we require that

$$ \langle I_{n}, Y \hat A_{F_{c}} + \hat A_{F_{c}}^{\mathsf{H}} Y\rangle +\langle {\Delta} \hat A_{F_{c}} + \hat A_{F_{c}}^{\mathsf{H}} {\Delta}, Y \hat A_{F_{c}} + \hat A_{F_{c}}^{\mathsf{H}} Y\rangle + 2\langle I_{n}, Y Q_{c} {\Delta}\rangle = 0 $$

for all $Y\in \mathbb {H}_{n}$. Using the properties of the scalar product, we obtain that this is equivalent to

$$ \langle Y, \hat A_{F_{c}}^{\mathsf{H}} + \hat A_{F_{c}} + \hat A_{F_{c}}{\Delta} \hat A_{F_{c}} + \hat A_{F_{c}} \hat A_{F_{c}}^{\mathsf{H}} {\Delta} + \hat A_{F_{c}}^{\mathsf{H}}{\Delta} \hat A_{F_{c}}^{\mathsf{H}} + {\Delta}\hat A_{F_{c}} \hat A_{F_{c}}^{\mathsf{H}} + Q_{c} {\Delta} + {\Delta} Q_{c} \rangle =0 $$

for all $Y\in \mathbb {H}_{n}$, or equivalently

19 $$  \hat A_{F_{c}}{\Delta} \hat A_{F_{c}} + \hat A_{F_{c}} \hat A_{F_{c}}^{\mathsf{H}} {\Delta} + \hat A_{F_{c}}^{\mathsf{H}}{\Delta} \hat A_{F_{c}}^{\mathsf{H}} + {\Delta}\hat A_{F_{c}} \hat A_{F_{c}}^{\mathsf{H}} + Q_{c} {\Delta} + {\Delta} Q_{c} = -\hat A_{F_{c}}^{\mathsf{H}}-\hat A_{F_{c}}. $$

If we fix a direction Δ and look for *α* such that *f*(*α*Δ) is minimal, then the one-dimensional Newton step corresponds to an inexact line search. It can be computed in an analogous way. With *g*(*α*) = *f*(*α*Δ), we then have

$$ g(\alpha)\approx f(0) + \alpha f_{X}(0)[{\Delta}] + \frac{1}{2} \alpha^{2}f_{XX}(0)[{\Delta}, {\Delta}] $$

and thus the one-dimensional Newton correction in *α* is given by

$$ \delta_{\alpha} = - \frac{\langle I_{n}, {\Delta} \hat A_{F_{c}} + \hat A_{F_{c}}^{\mathsf{H}} {\Delta} \rangle}{\langle I_{n}, {\Delta} Q_{c} {\Delta} \rangle + \frac{1}{2} \|{\Delta} \hat A_{F_{c}} + \hat A_{F_{c}}^{\mathsf{H}} {\Delta} \|^{2}_{F}}. $$

#### The Discrete-time Case

For the discrete-time case, we have that

$$ W_{d} (X_{0}+{\Delta}_{X}) = \left[ \begin{array}{cc} Q_{0} & C_{0}^{\mathsf{H}}\\ C_{0} & R_{0} \end{array} \right] - \left[\begin{array}{c} A^{\mathsf{H}} \\ B^{\mathsf{H}} \end{array} \right] {\Delta}_{X} \left[\begin{array}{cc} A & B \end{array} \right] + \left[\begin{array}{c} I_{n} \\ 0 \end{array} \right] {\Delta}_{X} \left[\begin{array}{cc} I_{n} & 0 \end{array} \right], $$

where

$$ \left[\begin{array}{cc} Q_{0} & C_{0}^{\mathsf{H}}\\ C_{0} & R_{0} \end{array}\right] := W_{d} (X_{0}). $$

By taking Schur complements and applying congruence transformations, it follows again that the product $\det W_{d} (X_{0}+{\Delta }_{X})(-1)^{n}$ is equal to
 where *R*_0_ = *R* − *B*^*H*^*X*_0_*B*, *C*_0_ = *C* − *B*^*H*^*X*_0_*A*, *Q*_0_ = *X*_0_ − *A*^*H*^*X*_0_*A*, $A_{F_{d}}:=A-BR_{0}^{-1}C_{0}^{\mathsf {H}}$ and $P_{0}:=Q_{0}-C_{0}R_{0}^{-1}C_{0}^{\mathsf {H}}$ are associated with the current point *X*_0_. Setting

$$ Z_{\ell} := \left[\begin{array}{cccc} P_{0}^{-\frac{1}{2}} & 0 & 0 & 0 \\ 0 & P_{0}^{\frac{1}{2}} & 0 & -\hat BR_{0}^{-1} \\ 0 & 0 & P_{0}^{-\frac{1}{2}}& 0 \\ 0 & 0 & 0 & R_{0}^{-\frac{1}{2}} \end{array}\right],\qquad Z_{r} := \left[\begin{array}{cccc} P_{0}^{\frac{1}{2}} & 0 & 0 & 0 \\ 0 & P_{0}^{-\frac{1}{2}} & 0 & 0 \\ 0 & 0 & P_{0}^{-\frac{1}{2}}& 0 \\ 0 & -R_{0}^{-1}\hat B^{\mathsf{H}}\hat {\Delta}_{X} & 0 & R_{0}^{-\frac{1}{2}} \end{array}\right], $$

transforming with *Z*_*ℓ*_ from the left and *Z*_*r*_ from the right, and substituting $\hat B = P_{0}^{\frac {1}{2}} B$, $\hat A_{F_{d}}:=P_{0}^{\frac {1}{2}}A_{F_{d}}P_{0}^{-\frac {1}{2}}$, and $\hat {\Delta }_{X}=P_{0}^{-\frac {1}{2}}{\Delta }_{X}P_{0}^{-\frac {1}{2}}$, we obtain the matrix

$$ \left[\begin{array}{cccc} -I_{n} & 0 & \hat {\Delta}_{X} & 0 \\ 0 & I_{n} -\hat BR_{0}^{-1}\hat B^{\mathsf{H}}\hat {\Delta}_{X} & \hat A_{F_{d}} & 0 \\ I_{n} & \hat A_{F_{d}}^{\mathsf{H}}\hat {\Delta}_{X} & I_{n} & 0 \\ 0 & 0 & 0 & I_{m} \end{array}\right] := Z_{\ell} \left[\begin{array}{cccc} -I_{n} & 0 & {\Delta}_{X} & 0 \\ 0 & I_{n} & A_{F_{d}} & B \\ I_{n} & A_{F_{d}}^{\mathsf{H}}{\Delta}_{X} & P_{0} & 0 \\ 0 & B^{\mathsf{H}}{\Delta}_{X} & 0 & R_{0} \end{array}\right] Z_{r}. $$

Using $\det Z_{\ell }\cdot \det Z_{r}= 1/(\det P_{0}\cdot \det R_{0})=1/\det W_{d}(X_{0})$ we obtain a similar lemma to the continuous-time case.

##### **Lemma 2**

The change of variables

$$ \hat B = P_{0}^{\frac{1}{2}} B, \quad \hat A_{F_{d}}:=P_{0}^{\frac{1}{2}}A_{F_{d}}P_{0}^{-\frac{1}{2}}, \quad \hat {\Delta}_{X}=P_{0}^{-\frac{1}{2}}{\Delta}_{X}P_{0}^{-\frac{1}{2}} $$

yields the following determinant identity

$$ \det W_{d}(X_{0}+{\Delta}_{X}) = \det \left[\begin{array}{cc} I_{n} -\hat BR_{0}^{-1}\hat B^{\mathsf{H}}\hat {\Delta}_{X} & \hat A_{F_{d}} \\ \hat A_{F_{d}}^{\mathsf{H}}\hat {\Delta}_{X} & I_{n} +\hat {\Delta}_{X} \end{array}\right]. $$

##### *Proof*

The proof is analogous to the continuous-time case. □

We have again an equivalent minimization problem $\min \limits _{X\in \mathbb {H}_{n}} f(X)$ in the “translated” variable $X=\hat {\Delta }_{X}$ with the barrier function

$$ \begin{array}{@{}rcl@{}} f(X)&:=& - \ln \Re \det (G(X)), \\ G(X)&:=& \left[\begin{array}{cc} I_{n} -Q_{d} X & \hat A_{F_{d}} \\ \hat A_{F_{d}}^{\mathsf{H}} X & I_{n} +X \end{array}\right], \\ Q_{d}&:=&\hat BR_{0}^{-1}\hat B^{\mathsf{H}}, \end{array} $$

and compute the gradient and the Hessian of *f*(*X*). The computation of the gradient is not as straight-forward as in the continuous-time case, since we consider non-Hermitian matrices. It is given by

$$ f_{X}(X)[{\Delta}] =\left\langle -\frac{\overline{\det G(X)}}{\Re\det G(X)}G(X)^{-\mathsf{H}}, \left[\begin{array}{cc} -Q_{d} {\Delta} & 0 \\ \hat A_{F_{d}}^{\mathsf{H}}{\Delta} & {\Delta} \end{array}\right]\right\rangle, $$

see Appendix A for more details. It follows from the derivation of *G*(*X*) in Lemma 2 that $\det (G(X))$ is positive and real and the solution of the minimization problem is still unique and Hermitian. Moreover, $\overline {\det G(X)}=\Re \det G(X)$ and the Hessian is then given by

$$ f_{XX}(X)[{\Delta},{\Delta}] =\left\langle G(X)^{-\mathsf{H}}\left[\begin{array}{cc} -Q_{d} {\Delta} & 0 \\ \hat A_{F_{d}}^{\mathsf{H}}{\Delta} & {\Delta} \end{array}\right]^{\mathsf{H}} G(X)^{-\mathsf{H}}, \left[\begin{array}{cc} -Q_{d} {\Delta} & 0 \\ \hat A_{F_{d}}^{\mathsf{H}}{\Delta} & {\Delta} \end{array}\right]\right\rangle, $$

and a second order approximation of *f* (at *X* = 0) is given by

$$ \begin{array}{@{}rcl@{}} f({\Delta}) &\approx& T^{(2)}_{f}({\Delta})\\ &=& f(0) +f_{X}(0)[{\Delta}] + \frac{1}{2}f_{XX}(0)[{\Delta}, {\Delta}]\\ &=& -\left\langle \begin{bmatrix} I_{n} & 0\\ -\hat A_{F_{d}}^{\mathsf{H}} & I_{n} \end{bmatrix}, \left[\begin{array}{cc} -Q_{d} Y & 0 \\ \hat A_{F_{d}}^{\mathsf{H}}Y & Y \end{array}\right]\right\rangle\\ &&+\frac{1}{2}\left\langle \begin{bmatrix} I_{n} & 0\\ -\hat A_{F_{d}}^{\mathsf{H}} & I_{n} \end{bmatrix} \left[\begin{array}{cc} -{\Delta} Q_{d} & {\Delta} \hat A_{F_{d}} \\ 0 & {\Delta} \end{array}\right] \begin{bmatrix} I_{n} & 0\\ -\hat A_{F_{d}}^{\mathsf{H}} & I_{n} \end{bmatrix}, \left[\begin{array}{cc} -Q_{d} {\Delta} & 0 \\ \hat A_{F_{d}}^{\mathsf{H}}{\Delta} & {\Delta} \end{array}\right]\right\rangle\\ &=& -\left\langle I_{n} - Q_{d} -\hat A_{F_{d}} \hat{A}_{F}^{\mathsf{H}}, {\Delta}\right\rangle\\ && +\frac{1}{2} \left\langle Q_{d}{\Delta} Q_{d}{\Delta} - 2\hat A_{F_{d}} \hat A_{F_{d}}^{\mathsf{H}} {\Delta} Q_{d} {\Delta} - \hat A_{F_{d}} \hat A_{F_{d}}^{\mathsf{H}} {\Delta} \hat A_{F_{d}} \hat A_{F_{d}}^{\mathsf{H}} {\Delta}\right.\\ &&\left.+ 2\hat A_{F_{d}} {\Delta} \hat A_{F_{d}}^{\mathsf{H}} {\Delta} - {\Delta}^{2}, I_{n}\right\rangle. \end{array} $$

We want the gradient of *f* to be 0, so for the Newton step we determine Δ = Δ^*H*^ such that $\frac {\partial T^{(2)}_{f}}{\partial {\Delta }}({\Delta })[Y]=0$ for all $Y\in \mathbb {H}_{n}$, or equivalently

$$ \begin{array}{@{}rcl@{}} 0&=&\langle I_{n} - Q_{d} -\hat A_{F_{d}} \hat A_{F_{d}}^{\mathsf{H}}, Y\rangle + \langle Q_{d}{\Delta} Q_{d} + \hat A_{F_{d}} \hat A_{F_{d}}^{\mathsf{H}} {\Delta} Q_{d}, Y \rangle\\ &&+ \langle Q_{d} {\Delta}\hat A_{F_{d}} \hat A_{F_{d}}^{\mathsf{H}} + \hat A_{F_{d}} \hat A_{F_{d}}^{\mathsf{H}} {\Delta} \hat A_{F_{d}} \hat A_{F_{d}}^{\mathsf{H}} - \hat A_{F_{d}} {\Delta} \hat A_{F_{d}}^{\mathsf{H}}- \hat A_{F_{d}}^{\mathsf{H}} {\Delta} \hat A_{F_{d}} + {\Delta}, Y\rangle \end{array} $$

for all $Y\in \mathbb {H}_{n}$. Using the properties of the scalar product, we obtain that

20 $$ \begin{array}{@{}rcl@{}} I_{n} - Q_{d} -\hat A_{F_{d}} \hat A_{F_{d}}^{\mathsf{H}} &=& Q_{d}{\Delta} Q_{d} + \hat A_{F_{d}} \hat A_{F_{d}}^{\mathsf{H}} {\Delta} Q_{d} +Q_{d} {\Delta}\hat A_{F_{d}} \hat A_{F_{d}}^{\mathsf{H}} + \hat A_{F_{d}} \hat A_{F_{d}}^{\mathsf{H}} {\Delta} \hat A_{F_{d}} \hat A_{F_{d}}^{\mathsf{H}} \\ && - \hat A_{F_{d}} {\Delta} \hat A_{F_{d}}^{\mathsf{H}} - \hat A_{F_{d}}^{\mathsf{H}} {\Delta} \hat A_{F_{d}} + {\Delta}. \end{array} $$

If we fix a direction Δ and look for *α* such that *f*(*α*Δ) is minimal, then the one-dimensional Newton step corresponds to an inexact line search. With *g*(*α*) = *f*(*α*Δ), we then have

$$ \delta_{\alpha} = \frac{2\left\langle I_{n} - Q_{d} -\hat A_{F_{d}} \hat{A}_{F}^{\mathsf{H}}, {\Delta}\right\rangle}{ \left\langle Q_{d}{\Delta} Q_{d}{\Delta} - 2\hat A_{F_{d}} \hat A_{F_{d}}^{\mathsf{H}} {\Delta} Q_{d} {\Delta} - \hat A_{F_{d}} \hat A_{F_{d}}^{\mathsf{H}} {\Delta} \hat A_{F_{d}} \hat A_{F_{d}}^{\mathsf{H}} {\Delta} + 2\hat A_{F_{d}} {\Delta} \hat A_{F_{d}}^{\mathsf{H}} {\Delta} - {\Delta}^{2},I_{n}\right\rangle}. $$

##### *Remark 4*

To carry out the Newton step, we have to solve equation (19) in the continuous-time case or (20) in the discrete-time case. This can be done via Kronecker products (for the cost of increasing the system dimension to *n*^2^), i.e., via

$$ \left( (I_{n} \otimes \hat{A}_{F_{c}} + \overline{\hat{A}_{F_{c}}} \otimes I_{n}) (\hat{A}_{F_{c}}^{T} \otimes I_{n} + I_{n} \otimes\hat{A}_{F_{c}}^{\mathsf{H}}) + I_{n} \otimes Q_{c} + \overline{Q}_{c} \otimes I_{n}\right) \text{vec} {\Delta} = \text{vec}(\hat{A}_{F_{c}} + \hat{A}_{F_{c}}^{\mathsf{H}}) $$

in the continuous-time case, or

$$ \begin{array}{@{}rcl@{}} &\left( (\overline{\hat{A}_{F_{d}}} \otimes \hat{A}_{F_{d}} - I_{n} \otimes I_{n})(\hat{A}_{F_{d}}^{T} \otimes \hat{A}_{F_{d}}^{\mathsf{H}} - I_{n} \otimes I_{n}) + \overline Q_{d} \otimes \hat{A}_{F_{c}}\hat{A}_{F_{c}}^{\mathsf{H}} + \overline{\hat{A}_{F_{c}}}\hat{A}_{F_{c}}^{T} \otimes Q_{d} + \overline{Q_{d}}\otimes Q_{d} \right) \text{vec} {\Delta}\quad\\ &\quad = \text{vec}(I_{n} -Q_{d} - \hat{A}_{F_{d}} \hat{A}_{F_{d}}^{\mathsf{H}}) \end{array} $$

in the discrete-time case.

#### Convergence

In this subsection, we show that the functions that we consider here actually have a globally converging Newton method. For this we have to analyze some more properties of our functions and refer to [6, 17] for more details.

Recall that a smooth function $f: \mathbb {R}^{n}\rightarrow \mathbb {R}$ is *self-concordant* if it is a closed and convex function with open domain and

$$ |f^{(3)}(x)| \le 2 \left( f^{(2)}(x)\right)^{\frac32} $$

in the case *n* = 1, and if *n* > 1, then *f* is *self-concordant* if it is self-concordant along every direction in its domain. In particular, if *n* = 1 then $f(x) = - \ln (x)$ is self-concordant and in general, if *f* is self-concordant and in addition $A\in \mathbb {C}^{n\times m}$, $b\in \mathbb {R}^{n}$, then *f*(*Ax* + *b*) is also self-concordant. These results can be easily extended to the real space of complex matrices showing that the function $b(X)= - \ln \det (W(X))$ is self-concordant. Let *b*_*X*_ and *b*_*XX*_ denote the gradient and the Hessian of the barrier function *b*(*X*), and let

$$ \lambda(X):= \left\langle (b_{XX})^{-1}b_{X}, b_{X}\right\rangle, $$

where Δ_*N*_ := (*b*_*XX*_)^− 1^*b*_*X*_ is the Newton step, i.e., $\lambda (X) = \langle {\Delta }_{N},P_{c}^{-1} A_{F_{c}}^{\mathsf {H}}+A_{F_{c}}P_{c}^{-1}\rangle $ in the continuous-time case, and $\lambda (X) = \langle {\Delta }_{N},A_{F_{d}}P_{d}^{-1}A_{F_{d}}^{\mathsf {H}} -P_{d}^{-1} + B(R-B^{\mathsf {H}}X B)^{-1}B^{\mathsf {H}}\rangle $ in the discrete-time case, respectively. In both cases *λ*(*X*) can be easily computed during the Newton step and gives an estimate of the residual of the current approximation of the solution.

Furthermore, note, that for every $X\in \mathbb {X}^{\succ \!\!\succ }_{c}~(\mathbb {X}^{\succ \!\!\succ }_{d})$ the incremental step Δ*W*(*X*)[Δ_*X*_] appearing in the directional derivative (11) is independent of *X*. Thus, the quadratic form of the Hessian can be expressed as

$$ \begin{array}{@{}rcl@{}} \langle b_{XX}{\Delta}_{X},{\Delta}_{X}\rangle &=& \langle W^{-1} {\Delta} W[{\Delta}_{X}] W^{-1}, {\Delta} W[{\Delta}_{X}]\rangle \\ &=& \text{tr}\left( W^{-\frac 12} {\Delta} W[{\Delta}_{X}] W^{-1} {\Delta} W[{\Delta}_{X}]W^{-\frac 12} \right). \end{array} $$

Using the Courant–Fischer theorem twice, see e.g. [3], this implies that

$$ \begin{array}{@{}rcl@{}} \text{tr}\left( W^{-\frac 12} {\Delta} W[{\Delta}_{X}] W^{-1} {\Delta} W[{\Delta}_{X}]W^{-\frac 12}\right) & \ge& \frac{1}{\lambda_{\max}(W(X))} \text{tr}\left( {\Delta} W[{\Delta}_{X}] W^{-1} {\Delta} W[{\Delta}_{X}]\right)\\ &\ge& \frac{1}{\lambda^{2}_{\max}(W(X))} \text{tr}\left( {\Delta} W[{\Delta}_{X}] {\Delta} W[{\Delta}_{X}]\right). \end{array} $$

Note that ∥Δ*W*[Δ_*X*_]∥_*F*_≠ 0 for controllable (*A*,*B*) and Δ_*X*_≠ 0. Minimizing the left-hand side over all Δ_*X*_ with $\|{\Delta }_{X}\|_{F}^{2}=1$ yields uniform positivity of the Hessian, since the spectrum of *W*(*X*) is bounded.

Hence, it follows, see e.g. [17, Theorem 4.1.14], that the Newton method is quadratically convergent, whenever *λ*(*X*) < .25 in some intermediate step. Once this level is reached, the methods stays in the quadratically convergent regime. If the condition does not hold, then one has to take a smaller stepsize $(1+\lambda (X))^{-1}{\Delta }_{X}$ in order to obtain convergence.

#### Initialization

Note that for the reformulations of the Newton step we have to assume that the starting value *X*_0_ is in the interior of the domain. In this section, we show how to compute an initial point $X_{0}\in \mathbb {X}^{\succ \!\!\succ }_{c}$ (or $X_{0}\in \mathbb {X}^{\succ \!\!\succ }_{d}$), which therefore satisfies the LMI $W_{c}(X_{0},{\mathscr{M}}) \succ 0$ (or $W_{d}(X_{0},{\mathscr{M}}) \succ 0$) for the model $\mathcal M = \{A,B,C,D\}$. Since the reasoning for both the continuous-time case and the discrete-time case are very similar, we first focus on the continuous-time case.

We start the optimization from a model ${\mathscr{M}}$ that is minimal and strictly passive. It then follows that the solution set of $W_{c}(X_{0},{\mathscr{M}}) \succ 0$ has an interior point *X*_0_ ≻ 0 such that

$$ W_{c}(X_{0},\mathcal{M}) \succ 0, \quad 0 \prec X_{-} \preceq X_{0} \preceq X_{+}, $$

where *X*_−_ and *X*_+_ are the Riccati solutions corresponding to this LMI. To construct such an *X*_0_, let $\alpha :=\lambda _{\min \limits }W_{c}(X_{0}) > 0$ and $\upbeta :=\max \limits (\|X_{0}\|_{2},1)>0$. Then, for 0 < 2*ξ* ≤ *α*/β, we have the inequality

21 $$  W_{c}(X_{0},\mathcal{M})- 2\xi \left[\begin{array}{cc} X_{0} & 0 \\ 0 & I_{m} \end{array}\right] \succeq 0. $$

In order to compute a solution *X*_0_ for this LMI, we rewrite it as

$$ W_{c}(X_{0},\mathcal{M}_{\xi}) := \left[\begin{array}{cc} -(A+\xi I_{n})^{\mathsf{H}}X_{0}-X_{0}(A+\xi I_{n}) &~C^{\mathsf{H}}-X_{0}B \\ C-B^{\mathsf{H}}X_{0} &~R - 2\xi I_{m} \end{array}\right] \succeq 0 $$

for the modified model ${\mathscr{M}}_{\xi } :=\{A+\xi I_{n},B,C,D-\xi I_{m}\}$ and *R* = *D* + *D*^*H*^. It then follows from (21) that ${\mathscr{M}}_{\xi }$ is passive. Therefore we have the following lemma.

##### **Lemma 3**

Let ${\mathscr{M}}:=\{A,B,C,D\}$ be strictly passive. Then there exists a sufficiently small *ξ* > 0 such that the modified model ${\mathscr{M}}_{\xi } :=\{A+\xi I_{n},B,C,D-\xi I_{m}\}$ is passive. Then the extremal solutions *X*_−_(*ξ*) and *X*_+_(*ξ*) of the model ${\mathscr{M}}_{\xi }$ are interior points of $\mathbb {X}^{\succ \!\!\succ }_{c}$.

##### *Proof*

Let *X*_0_ be any point such that $W_{c}(X_{0},{\mathscr{M}}_{\xi })\succeq 0$ and *ξ* > 0. Then it follows from (21) that $W_{c}(X_{0},{\mathscr{M}})\succ 0$ and hence it is an interior point of $\mathbb {X}^{\succ \!\!\succ }_{c}$. This also applies to the Riccati solutions *X*_−_(*ξ*) and *X*_+_(*ξ*). □

The reasoning for the discrete-time case is very similar. Starting from a strictly passive and minimal model ${\mathscr{M}}$, we have the inequality

$$ W_{d}(\mathcal{M}) - 2\xi \left[\begin{array}{cc} X_{0} & 0 \\ 0 & I_{m} \end{array}\right] \succeq 0 \quad \mathrm{ for }~0< 2\xi \le \alpha/\upbeta=\lambda_{\min}W_{d}(X_{0})/\max(\|X_{0}\|_{2},1). $$

In order to compute a solution *X*_0_ for this LMI, we rewrite it as the scaled LMI

$$ W_{d}(X_{0},\mathcal{M}_{\xi}) := (1-2\xi)\left[\begin{array}{cc} X_{0}-A_{\xi}^{\mathsf{H}}X_{0}A_{\xi} & C_{\xi}^{\mathsf{H}}-A_{\xi}^{\mathsf{H}} X_{0}B_{\xi} \\ C_{\xi}-B_{\xi}^{\mathsf{H}}X_{0} A_{\xi} &R_{\xi} -B_{\xi}^{\mathsf{H}} X_{0}B_{\xi} \end{array}\right] \succeq 0 $$

for the modified model ${\mathscr{M}}_{\xi } :=\{A_{\xi },B_{\xi },C_{\xi },D_{\xi } \} := \{A/\sqrt {1-2\xi },B/\sqrt {1-2\xi },C/(1-2\xi ), (D-\xi I_{m})/(1-2\xi ) \}$ and $R_{\xi } = D_{\xi } + D_{\xi }^{\mathsf H}$. The solutions *X*_−_(*ξ*) and *X*_+_(*ξ*) of this scaled LMI are again strictly included in the original solution set.

The procedure to find an inner point is thus to choose one of the Riccati solutions *X*_−_(*ξ*) or *X*_+_(*ξ*) of shifted or scaled problems, respectively, or some kind of average of both, since they are then guaranteed to be an interior point of the original problem. An upper bound for 2*ξ* is $\lambda _{\min \limits }(R)$. If the Riccati solutions of ${\mathscr{M}}_{\xi }$ indicate that the shifted model is not passive, *ξ* is divided by 2.

Another possibility to compute an initial point is to take the *geometric mean* of the minimal and maximal solution of the Riccati equations (7), respectively (10), denoted by *X*_−_ and *X*_+_, which is defined by $X_{0} = X_{-} (X_{-}^{-1}X_{+})^{\frac {1}{2}}$, see [16]. However, e.g., if *X*_−_ and *X*_+_ are multiples of the identity matrix, then the geometric mean is a convex combination of *X*_−_ and *X*_+_ and will not necessarily be in the interior.

### Numerical Results

We have implemented the steepest descent method of Section 4.1 and the Newton method introduced in Section 4.2. The software package is written in python 3.6. The code and all the examples can be downloaded under [1].

We have performed several experiments to test convergence for the different methods developed in this paper. All of them present qualitatively similar convergence behavior.

#### *Example 1*

As a prototypical example consider a randomly generated continuous-time example with real coefficients and *n* = 30 and *m* = 10, i.e., the overall dimension of the matrix *W*_*c*_(*X*) is 40 × 40 and we have a total of 465 unknowns.

As one would expect, the steepest descent method shows linear convergence behavior, whereas the Newton method has quadratic convergence as soon as one is close enough to the analytic center.

**Fig. 1:**
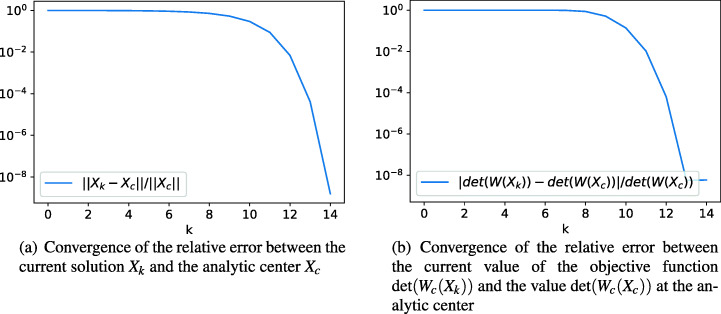
Convergence behavior for the Newton method applied to Example 1

Figure 1 shows the convergence behavior using the Newton method. The number of steps required in the steepest descent approach, however, is much higher than in the Newton approach. Table 1 shows the convergence behavior of the steepest descent method after starting the algorithm at a point well inside the feasible region, which has been obtained from a previous run with the Newton method. One can see, that even after 10,000 steps, there is no significant improvement for the residual in the determinant of *W*(*X*). Though, one can at least confirm, that the values are monotonously decreasing as expected.

**Table 1:** Convergence of the relative error of the current value of the objective function $\det (W_{c}(X_{k}))$ and the intermediate solutions *X*_*k*_ for the steepest descent method applied to Example 1

*k*	1	10	100	1,000	10,000
$\frac {|\det (W_{c}(X_{k}))-\det (W_{c}(X_{c}))|}{\det (W_{c}(X_{c}))}$	0.86808524	0.86808522	0.86808512	0.86808512	0.86808428
$\frac {\|X_{k}-X_{c}\|}{\|X_{c}\|}$	0.72171198	0.72171198	0.72171197	0.72171194	0.7217116

Also, the initial point computed by the geometric mean approach turns out to be much better in all the practical examples, even though one cannot guarantee positivity in some extreme cases.

Note that one has to be extremely careful with the implementation of the algorithm. Without explicitly forcing the intermediate solutions *X*_*k*_ to be Hermitian in finite precision arithmetic, the intermediate Riccati residuals *P*_*k*_ may diverge from the Hermitian subspace.

## Computation of Bounds for the Passivity Radius

Once we have found a solution $X\in \mathbb {X}^{\succ \!\!\succ }_{c}$, respectively $X\in \mathbb {X}^{\succ \!\!\succ }_{d}$, we can use this solution to find an estimate of the *passivity radius* of our system, i.e., the smallest perturbation ${\Delta }_{{\mathscr{M}}}$ to the system coefficients ${\mathscr{M}}=\{A,B,C,D\}$ that puts the system on the boundary of the set of passive systems, so that an arbitrary small further perturbation makes the system non-passive. In this section we derive *lower bounds* for the passivity radius in terms of the smallest eigenvalue of a scaled version of the matrices $W_{c}(X,{\mathscr{M}})$ or $W_{d}(X,{\mathscr{M}})$, respectively. Since the analytic center is central to the solution set of the LMI, we choose it for the realization of the transfer function, since then we expect to maximize a very good lower bound for the passivity radius.

### The Continuous-time Case

As soon as we fix $X\in \mathbb {X}^{\succ \!\!\succ }_{c}$, the matrix

$$ W_{c}(X,\mathcal{M}) = \left[ \begin{array}{cc} -A^{\mathsf{H}}X - X A &~C^{\mathsf{H}}-X B \\ C-B^{\mathsf{H}}X &~D+D^{\mathsf{H}} \end{array}\right] $$

is linear as a function of the coefficients *A*, *B*, *C*, *D*. When perturbing the coefficients, we thus preserve strict passivity, as long as

$$ W_{c}(X,\mathcal{M}+{\Delta}_{\mathcal{M}}) := \left[ \begin{array}{cc} -(A+{\Delta}_{A})^{\mathsf{H}}X - X (A+{\Delta}_{A}) &~(C+{\Delta}_{C})^{\mathsf{H}}-X (B+{\Delta}_{B})) \\ (C+{\Delta}_{C})-(B+{\Delta}_{B})^{\mathsf{H}}X &~(D+{\Delta}_{D}) +(D+{\Delta}_{D})^{\mathsf{H}} \end{array} \right] \succ 0. $$

We thus suppose that $W_{c}(X,{\mathscr{M}}) \succ 0$ and look for the smallest perturbation ${\Delta }_{{\mathscr{M}}}$ to our model ${\mathscr{M}}$ that makes $\det W_{c}(X,{\mathscr{M}}+{\Delta }_{{\mathscr{M}}})=0$. To measure the model perturbation, we propose to use the norm of the perturbation of the system pencil

$$ \|{\Delta}_{\mathcal{M}}\| := \left \|\left[\begin{array}{ccc} 0 & {\Delta}_{A} & {\Delta}_{B} \\ {\Delta}_{A}^{\mathsf{H}} & 0 & {\Delta}_{C}^{\mathsf{H}} \\ {\Delta}_{B}^{\mathsf{H}} & {\Delta}_{C} & {\Delta}_{D}+{\Delta}_{D}^{\mathsf{H}} \end{array}\right] \right\|_{2} \approx \left \|\left[\begin{array}{ccc} {\Delta}_{A} & {\Delta}_{B} \\ {\Delta}_{C} & {\Delta}_{D} \end{array}\right] \right \|_{2} $$

which holds when Δ_*D*_ is Hermitian and where ∥⋅∥_2_ denotes the matrix 2-norm. We have the following lower bound in terms of the smallest eigenvalue $\lambda _{\min \limits }$ of a scaled version of $W_{c}(X,{\mathscr{M}})$.

#### **Lemma 4**

The *X-passivity radius*, defined for a given $X\in \mathbb {X}^{\succ \!\!\succ }_{c}$ as

$$ \rho_{\mathcal{M}}^{c}(X):= \inf_{{\Delta}_{\mathcal{M}}}\{\|{\Delta}_{\mathcal{M}}\| ~|~ \det W_{c}(X,\mathcal{M}+{\Delta}_{\mathcal{M}}) = 0\}, $$

satisfies

22 $$  \lambda_{\min}(Y_{c} W_{c}(X,\mathcal{M}) Y_{c}) \le \rho_{\mathcal{M}}^{c}(X), $$

for

$$ Y_{c}:= \left[\begin{array}{cc} I_{n}+X^{2} & 0 \\ 0 & I_{m} \end{array}\right]^{-\frac{1}{2}}\preceq I_{n+m}. $$

#### *Proof*

We first note that

$$ \det \left[\begin{array}{cccc} 0 & I_{n} & X & 0 \\ I_{n} & 0 & A+{\Delta}_{A} & B+{\Delta}_{B} \\ X & A^{\mathsf{H}}+{\Delta}_{A}^{\mathsf{H}} & 0 & C^{\mathsf{H}}+{\Delta}_{C}^{\mathsf{H}} \\ 0 & B^{\mathsf{H}}+{\Delta}_{B}^{\mathsf{H}} & C+{\Delta}_{C} & R+{\Delta}_{R}^{\mathsf{H}} \end{array}\right] = \det \left[\begin{array}{cc} 0 & I_{n} \\ I_{n} & 0 \end{array}\right] \det W_{c}(X,\mathcal{M}+{\Delta}_{\mathcal{M}}), $$

since $W_{c}(X,{\mathscr{M}}+{\Delta }_{{\mathscr{M}}})$ is just the Schur complement with respect to the leading 2*n* × 2*n* matrix. Here we have set *R* := *D* + *D*^*H*^ and ${\Delta }_{R}:= {\Delta }_{D}+{\Delta }_{D}^{\mathsf {H}}$.

If we introduce the *n* × (*n* + *m*) matrix $Z_{c} := \left [\begin {array}{cc} -X & 0 \end {array}\right ]$, then it follows that

$$ \left[\begin{array}{c} Z_{c} \\ I_{m+n} \end{array}\right]^{\mathsf{H}} \left[\begin{array}{ccc} 0 &~A+{\Delta}_{A} &~B+{\Delta}_{B} \\ A^{\mathsf{H}}+{\Delta}_{A}^{\mathsf{H}} &~0 &~C^{\mathsf{H}}+{\Delta}_{C}^{\mathsf{H}} \\ B^{\mathsf{H}}+{\Delta}_{B}^{\mathsf{H}} &~C+{\Delta}_{C} &~R+{\Delta}_{R} \end{array}\right] \left[\begin{array}{c} Z_{c} \\ I_{m+n} \end{array}\right] = W_{c}(X, \mathcal{M}+{\Delta}_{\mathcal{M}}). $$

If we replace the matrix $\left [\begin {array}{c} Z_{c} \\ I_{m+n} \end {array}\right ]$ by the matrix $U_{c} = \left [\begin {array}{c} Z_{c} \\ I_{m+n} \end {array}\right ]Y_{c}$ with orthonormal columns, which we can e.g. obtain from a QR decomposition [11], then we obtain

$$ \begin{array}{@{}rcl@{}} U_{c}^{\mathsf{H}} \left[\begin{array}{ccc} 0 & A+{\Delta}_{A} & B+{\Delta}_{B} \\ A^{\mathsf{H}}+{\Delta}_{A}^{\mathsf{H}} & 0 & C^{\mathsf{H}}+{\Delta}_{C}^{\mathsf{H}} \\ B^{\mathsf{H}}+{\Delta}_{B}^{\mathsf{H}} & C+{\Delta}_{C} & R+{\Delta}_{R} \end{array}\right] U_{c} &=& Y_{c}W_{c}(X,\mathcal{M}+{\Delta}_{\mathcal{M}})Y_{c} \\ &=& Y_{c} W_{c}(X,\mathcal{M}) Y_{c} + U_{c}^{\mathsf{H}}\left[\begin{array}{ccc} 0 & {\Delta}_{A} & {\Delta}_{B} \\ {\Delta}_{A}^{\mathsf{H}} & 0 & {\Delta}_{C}^{\mathsf{H}} \\ {\Delta}_{B}^{\mathsf{H}} & {\Delta}_{C} & {\Delta}_{R} \end{array}\right]U_{c}. \end{array} $$

Therefore, the smallest perturbation of the matrix $ Y_{c} W_{c}(X,{\mathscr{M}}) Y_{c}$ to make $Y_{c}W_{c}(X,{\mathscr{M}}+{\Delta }_{{\mathscr{M}}})Y_{c}$ singular must have a 2-norm which is at least as large as $\lambda _{\min \limits }(Y_{c} W_{c}(X,{\mathscr{M}}) Y_{c})$, and since the norm of the second term in the right hand side is bounded by $\|{\Delta }_{{\mathscr{M}}}\|$, the lower bound in (22) follows. □

### The Discrete-time Case

In the discrete-time case, for a fixed *X* the LMI takes the form

$$ W_{d}(X) = \left[\begin{array}{cc} -A^{\mathsf{H}}XA + X & C^{\mathsf{H}} -A^{\mathsf{H}}XB \\ C-B^{\mathsf{H}}XA & D+D^{\mathsf{H}}-B^{\mathsf{H}}XB \end{array}\right] \succeq 0, $$

and its perturbed version is

$$ \begin{array}{@{}rcl@{}} &&W_{d}(X,\mathcal{M}+{\Delta}_{\mathcal{M}}) \\ && \quad :=\left[\begin{array}{cc} -(A+{\Delta}_{A})^{\mathsf{H}}X(A+{\Delta}_{A})+ X & (C+{\Delta}_{C})^{\mathsf{H}}- (A+{\Delta}_{A})^{\mathsf{H}}X(B+{\Delta}_{B}) \\ C+{\Delta}_{C}-(B+{\Delta}_{B})^{\mathsf{H}}X(A+{\Delta}_{A}) & R+{\Delta}_{R} -(B+{\Delta}_{B})^{\mathsf{H}}X(B+{\Delta}_{B}) \end{array}\right]\\ && \quad \succ 0, \end{array} $$

where again *R* := *D* + *D*^*H*^ and ${\Delta }_{R}:= {\Delta }_{D}+{\Delta }_{D}^{\mathsf {H}}$.

Note that, in contrast to the continuous-time case, for given $X\in \mathbb {X}^{\succ \!\!\succ }_{d}$, $W_{d}(X,{\mathscr{M}}+{\Delta }_{{\mathscr{M}}})$ is not linear in the perturbations. Nevertheless, we have an analogous bound as in Lemma 4 also in the discrete-time case.

#### **Lemma 5**

The *X-passivity radius*, defined for a given $X\in \mathbb {X}^{\succ \!\!\succ }_{d}$ as

$$ \rho_{\mathcal{M}}^{d}(X):= \inf_{{\Delta}_{\mathcal{M}}}\{\|{\Delta}_{\mathcal{M}}\|~ |~\det W_{d}(X,\mathcal{M}+{\Delta}_{\mathcal{M}}) = 0\}, $$

satisfies

$$ \lambda_{\min}\left( Y_{d} \left( W_{d}(X,\mathcal{M}) - \left[\begin{array}{cc} A^{\mathsf{H}} +I_{n} \\ B^{\mathsf{H}} \end{array}\right]\frac{X}{2}\left[\begin{array}{cc} {\Delta}_{A} & {\Delta}_{B} \end{array}\right] - \left[\begin{array}{c} {\Delta}_{A}^{\mathsf{H}}\\ {\Delta}_{B}^{\mathsf{H}} \end{array} \right]\frac{X}{2}\left[\begin{array}{cc} A +I_{n} & B \end{array}\right]\right) Y_{d}\right) \le \rho^{d}_{\mathcal{M}}(X), $$

where

$$ Y_{d}:= \left[\begin{array}{cc} I_{n+m} + Z_{d}^{\mathsf{H}}Z_{d} \end{array}\right]^{-\frac{1}{2}}\preceq I_{n+m}, \qquad Z_{d} :=-\left[\begin{array}{cc} \frac{X}{2}(A+{\Delta}_{A}-I_{n}) & \frac{X}{2}(B+{\Delta}_{B}) \end{array}\right]. $$

#### *Proof*

We first observe that

23 $$ \begin{array}{@{}rcl@{}} &&\det \left[\begin{array}{cccc} 0 &~I_{n} &~\frac{X}{2}(A+{\Delta}_{A}-I_{n}) &~\frac{X}{2}(B+{\Delta}_{B}) \\ I_{n} &~0 &~A+{\Delta}_{A}+I_{n} &~B+{\Delta}_{B} \\ (A^{\mathsf{H}}+{\Delta}_{A}^{\mathsf{H}}-I_{n})\frac{X}{2} &~A^{\mathsf{H}}+{\Delta}_{A}^{\mathsf{H}}+I_{n} &~0 &~C^{\mathsf{H}}+{\Delta}_{C}^{\mathsf{H}} \\ (B^{\mathsf{H}}+{\Delta}_{B}^{\mathsf{H}})\frac{X}{2} &~B^{\mathsf{H}}+{\Delta}_{B}^{\mathsf{H}} &~C+{\Delta}_{C} &~R+{\Delta}_{R} \end{array}\right]\\ && \quad = \det \left[\begin{array}{cc} 0 & I_{n} \\ I_{n} & 0 \end{array}\right] \det W_{d}(X,\mathcal{M}+{\Delta}_{\mathcal{M}}), \end{array} $$

since again $W_{d}(X,{\mathscr{M}}+{\Delta }_{{\mathscr{M}}})$ is just the Schur complement with respect to the leading 2*n* × 2*n* matrix. Note that this matrix (23) is linear in the perturbation parameters, since *X* is fixed. Using the definition of the matrix *Z*_*d*_, then from (23), it follows that

$$ \left[\begin{array}{cc} Z_{d}^{\mathsf{H}} &~I_{m+n} \end{array}\right] \left[\begin{array}{ccc} 0 &~A+{\Delta}_{A}+I_{n} &~B+{\Delta}_{B} \\ A^{\mathsf{H}}+{\Delta}_{A}^{\mathsf{H}}+I_{n} &~0 &~C^{\mathsf{H}}+{\Delta}_{C}^{\mathsf{H}} \\ B^{\mathsf{H}}+{\Delta}_{B}^{\mathsf{H}} &~C+{\Delta}_{C} &~R+{\Delta}_{R} \end{array}\right] \left[\begin{array}{c} Z_{d} \\ I_{m+n} \end{array}\right] = W_{d}(X,\mathcal{M}+{\Delta}_{\mathcal{M}}). $$

If we replace the matrix $\left [\begin {array}{c} Z_{d}\\ I_{m+n} \end {array}\right ]$ by the matrix with orthonormal columns $U_{d} = \left [\begin {array}{c} Z_{d} \\ I_{m+n} \end {array}\right ]Y_{d}$, then we have

$$ U_{d}^{\mathsf{H}} \left[\begin{array}{ccc} 0 &~A+{\Delta}_{A}+I_{n} &~B+{\Delta}_{B} \\ A^{\mathsf{H}}+{\Delta}_{A}^{\mathsf{H}}+I_{n} &~0 &~C^{\mathsf{H}}+{\Delta}_{C}^{\mathsf{H}} \\ B^{\mathsf{H}}+{\Delta}_{B}^{\mathsf{H}} &~C+{\Delta}_{C} &~R+{\Delta}_{R} \end{array}\right] U_{d} = Y_{d} W_{d}(X,\mathcal{M}+{\Delta}_{\mathcal{M}})Y_{d} $$

from which it follows that

$$ \begin{array}{@{}rcl@{}} &&Y_{d} W_{d}(X,\mathcal{M}+{\Delta}_{\mathcal{M}})Y_{d} = U_{d}^{\mathsf{H}} \left[\begin{array}{ccc} 0 &~{\Delta}_{A} &~{\Delta}_{B} \\ {\Delta}_{A}^{\mathsf{H}} &~0 &~{\Delta}_{C}^{\mathsf{H}} \\ {\Delta}_{B}^{\mathsf{H}} &~{\Delta}_{C} &~{\Delta}_{R} \end{array}\right] U_{d} \\ &&\qquad + Y_{d} \left( W_{d}(X,\mathcal{M})- \left[\begin{array}{cc} A^{\mathsf{H}} +I_{n} \\ B^{\mathsf{H}} \end{array}\right]\frac{X}{2}\left[\begin{array}{cc} {\Delta}_{A} & {\Delta}_{B} \end{array}\right] - \left[\begin{array}{c} {\Delta}_{A}^{\mathsf{H}}\\ {\Delta}_{B}^{\mathsf{H}} \end{array} \right]\frac{X}{2}\left[\begin{array}{cc} A +I_{n} & B \end{array}\right] \right) Y_{d}, \end{array} $$

and using the same argument as for the continuous-time case, it follows that the smallest perturbation of the matrix $ Y_{d} W_{d}(X,{\mathscr{M}}) Y_{d}$ needed to make $Y_{d}W_{d}(X,{\mathscr{M}}+{\Delta }_{{\mathscr{M}}})Y_{d}$ singular must have a 2-norm which is at least as large as

$$ \lambda_{\min} \left( Y_{d} \left( W_{d}(X,\mathcal{M})- \left[\begin{array}{cc} A^{\mathsf{H}} +I_{n} \\ B^{\mathsf{H}} \end{array}\right]\frac{X}{2}\left[\begin{array}{cc} {\Delta}_{A} & {\Delta}_{B} \end{array}\right] - \left[\begin{array}{c} {\Delta}_{A}^{\mathsf{H}}\\ {\Delta}_{B}^{\mathsf{H}} \end{array} \right]\frac{X}{2}\left[\begin{array}{cc} A +I_{n} &~B \end{array}\right] \right) Y_{d}\right). $$

□

### Examples with Analytic Solution

In this subsection, to illustrate the results, we present simple examples of scalar transfer functions (*m* = 1) of first degree (*n* = 1).

Consider first an asymptotically stable continuous-time system and transfer function $T(s)=d+\frac {cb}{s-a}$, i.e., with *a* < 0. Then

$$ W_{c}(x) = \left[\begin{array}{cc} -2ax &~c-bx \\ c-bx &~2d \end{array}\right] $$

and its determinant is $\det (W_{c}(x)) = -4adx-(c-bx)^{2}$, which is maximal at the central point $x_{a}=\frac {c}{b}-\frac {2ad}{b^{2}}$. We then get

$$ W_{c}(x_{a}) = \left[\begin{array}{cc} 4d\frac{a^{2}}{b^{2}}-2c\frac{a}{b} &~2d\frac{a}{b} \\ 2d\frac{a}{b} &~2d \end{array}\right] = \left[\begin{array}{cc} 1 & \frac{a}{b} \\ 0 & 1 \end{array}\right]\cdot \left[\begin{array}{cc}p & 0 \\ 0 & 2d \end{array}\right] \left[\begin{array}{cc} 1 & 0 \\ \frac{a}{b} & 1 \end{array}\right], $$

with $p=2d\frac {a^{2}}{b^{2}}-2c\frac {a}{b}$ which implies that $\det (W_{c}(x_{a}))=2d \cdot p$. For the transfer function to be strictly passive, it must be asymptotically stable and positive on the imaginary axis and hence also at 0 and $\infty $. Thus, we have the conditions

24 $$  a < 0,\quad d > 0, \quad \frac{da-cb}{a} > 0. $$

The function ${\Phi }_{c}(\imath \omega )=2d - \frac {2acb}{a^{2}+\omega ^{2}}$ is a unimodal function, which reaches its minimum either at 0 (namely ${\Phi }_{c}(0)=p\frac {b^{2}}{a^{2}}$) or at $\infty $ (namely ${\Phi }_{c}(\infty )=2d$) and hence the conditions in (24) are sufficient to check passivity. Thus, for the model ${\mathscr{M}}$, strict passivity gets lost when either one of the following happens

$$ d+\delta_{d}=0, \quad a+\delta_{a} = 0, \quad \left[ \begin{array}{cc} c+\delta_{c} &~d+\delta_{d} \end{array} \right] \left[ \begin{array}{c} -b-\delta_{b} \\ a+\delta_{a} \end{array} \right]=0. $$

Therefore, it follows that

$$ \rho = \min\left( d, a, \sigma_{2} \left[ \begin{array}{ccc} a & b \\ c & d \end{array} \right]\right)= \sigma_{2} \left[ \begin{array}{ccc} a & b \\ c & d \end{array} \right] $$

At the analytic center *x*_*a*_ we have

$$ \det W_{c}(x_{a})=2dp= 4\frac{ad}{b^{2}}(ad-bc) $$

and the smallest perturbation of the parameters that makes this determinant go to 0, yields exactly the same conditions as (24). This illustrates that the *X*-passivity radius at the analytic center yields a very good condition for strict passivity of the model.

In the discrete-time case the transfer function is $T(z)=d+\frac {cb}{z-a}$ and for it to be asymptotically stable we need *a*^2^ < 1, when we assume the coefficients to be real. Then

$$ W_{d}(x) = \left[\begin{array}{cc} x- a^{2}x &~c -abx\\ c - abx &~2d - b^{2}x \end{array}\right] $$

and the analytic center, where $\det W_{d}(x) = (1-a^{2})x(2d-b^{2}x)-(c-abx)^{2}$ is maximal, is given by $x_{a}=\frac {d-a^{2}d+a b c}{b^{2}}$ with

$$ \det W_{d}(x_{a})=\frac{(a^{2}-1) (bc-(a-1)d) (b c-(a+1)d)}{b^{2}}. $$

The function ${\Phi }_{d}(z) = \frac {b c}{\frac {1}{z}-a}+\frac {b c}{z-a}+2 d$ will be minimal on the unit circle at *z* = 1 or *z* = − 1. Thus positivity will be lost, when either *a* reaches 1 or − 1, or *bc* − (*a* − 1)*d* = 0 or *bc* − (*a* + 1)*d* = 0. This is exactly the condition also reflected in the determinant of *W*(*x*_*c*_) at the analytic center *x*_*a*_. This again illustrates that the *X*-passivity radius at the analytic center gives a good bound the passivity radius of the system.

## Concluding Remarks

We have derived conditions for the analytic center of the linear matrix inequalities (LMIs) associated with the passivity of linear continuous-time or discrete-time systems. We have presented numerical methods to compute these analytic centers with steepest descent and Newton methods and we have presented lower bounds for the passivity radii associated with the LMIs evaluated at the respective analytic center.

## Appendix A:: Derivatives of Functions of Complex Matrices

In this appendix, we present a precise derivation of the formulas for the differentiation of a matrix function with respect to a complex matrix. Here, we distinguish between complex vector spaces $\mathbb {C}^{n}$ and the corresponding real vector space $\mathbb {R}^{n} + \imath \mathbb {R}^{n}$. Both spaces can be identified by $\mathbf {c}: \mathbb {C}^{n} \rightarrow \mathbb {R}^{n} + \imath \mathbb {R}^{n}$, **c**(*v*) = *R*(*v*) + *ı**I*(*v*). For matrix spaces of dimension *m* × *n* we use the usual identification with the vector spaces $\mathbb {C}^{n}$ and $\mathbb {R}^{n} + \imath \mathbb {R}^{n}$. The space $\mathbb {C}^{n}$ is equipped with the standard scalar product $\langle x,y \rangle _{\mathbb {C}}:= x^{\mathsf {H}}y$. By $\frac {\partial }{\partial X}$ we denote the differentiation in a real vector space, whereas the differentiation of a holomorphic function *g* is denoted by *g*^′^. Note that if we write **c** ∘ *g*(*x*) = *u*(*x*_*r*_ + *ı**x*_*i*_) + *ı**v*(*x*_*r*_ + *ı**x*_*i*_), then by the Cauchy–Riemann equations, see e.g. [9], we have $\mathbf c \circ g^{\prime }(x) = \frac {\partial }{\partial x_{r}} u(x_{r} + \imath x_{i})- \imath \frac {\partial }{\partial x_{i}} u(x_{r} + \imath x_{i})$. Then we have the following result:

### **Lemma 6**

Assume that $g: \mathbb {C}^{n\times n} \rightarrow \mathbb {C}$ is holomorphic. Then $f: \mathbb {R}^{n\times n} + \imath \mathbb {R}^{n\times n} \rightarrow \mathbb {R}$ defined by

$$ f(X_{r} + \imath X_{i}) : = \Re g(X) $$

is differentiable over $\mathbb R$ with

$$ \frac{\partial}{\partial X} f(X_{r} + \imath X_{i}) = \Re (\overline{g^{\prime}(X)} \circ \mathbf{c^{-1}}) $$

and

$$ \left\langle \frac{\partial}{\partial X} f(X_{r} + \imath X_{i}), {\Delta} \right\rangle_{\mathbb{R}} = \Re \langle \overline{g^{\prime}(X)}, \mathbf{c^{-1}}({\Delta}) \rangle_{\mathbb{C}}, \quad {\Delta} = {\Delta}_{r} + \imath {\Delta}_{i}. $$

For the holomorphic function $g(X) = \det (X)$ the following fact is well-known, see e.g. [14] for a proof in the real case, that easily extends to the complex case.

### **Lemma 7**

(Jacobi’s formula) Let $g(X) = \det (X)$ and $X\in \mathbb {C}^{n\times n}$. Then $g^{\prime }(X)=\text {adj}(X^{T})$ and the directional derivative of *g* in the direction ${\Delta }\in \mathbb {C}^{n\times n}$ equals

$$ g^{\prime}(X)\circ{\Delta} = \text{tr}(\text{adj}(X){\Delta}) = \langle \text{adj}(X)^{\mathsf{H}}, {\Delta} \rangle_{\mathbb{C}}. $$

Applying the chain-rule we finally obtain the differentiation formula, which is used throughout this paper.

### **Corollary 1**

Let $f: \mathbb {R}^{n\times n} + \imath \mathbb {R}^{n\times n} \rightarrow \mathbb {R}$ with $f(X_{r} + \imath X_{i}) = \ln \Re \det (X)$ and $X\in \mathbb {C}^{n\times n}$ with $\Re \det (X)>0$. Then

$$ \frac{\partial}{\partial X} f(X_{r} + \imath X_{i}) = \mathbf c \circ \left( \frac{\overline{\det(X)}}{\Re\det(X)}X^{-\mathsf H}\right). $$

Moreover, if $X\in \mathbb {H}_{n}$ then

$$ \frac{\partial}{\partial X} f(X_{r} + \imath X_{i}) = \mathbf c \circ (X^{-\mathsf H}). $$

## Appendix B:: Differences Between Continuous-time and Discrete-time Systems

Usually, statements for a continuous linear time-invariant system can be transformed back and forth to discrete-time systems using some bilinear transform. However, the equations determining the analytic center in both cases are cubic in *X*, which suggests that there might not be a one-to-one correspondence. We have shown that the eigenvalues of the feedback system matrix $A_{F_{c}}$ at the analytic center lie on the imaginary axis in the continuous-time case, whereas they lie inside the unit disk in the discrete-time setting. In this appendix we show that it is indeed necessary to consider the continuous-time and discrete-time case separately by showing that the three equations determining the analytic center are not preserved under the usual bilinear transformations. In the first subsection we show that the domains of the LMI’s are the same for the continuous-time and discrete-time cases, but in the second subsection we show that the feedback associated with the analytic center are not related via a bilinear transformation.

### B.1 Bilinear Transformations

The bilinear transformation *s* = (*z* − 1)/(*z* + 1) maps every asymptotically stable continuous-time system {*A*_*c*_,*B*_*c*_,*C*_*c*_,*D*_*c*_} to a corresponding asymptotically stable discrete-time system {*A*_*d*_,*B*_*d*_,*C*_*d*_,*D*_*d*_}.

Let us start with an asymptotically stable continuous-time system {*A*_*c*_,*B*_*c*_,*C*_*c*_,*D*_*c*_}. For some $Q_{c}\in \mathbb {C}^{n\times n}$ and $R_{c} = D_{c} + D_{c}^{\mathsf {H}}$ set

25 $$ Z_{c} := \left[ \begin{array}{cc} \sqrt{2}(I-A_{c})^{-1} &~(I-A_{c})^{-1}B_{c}\\ 0 &~I \end{array}\right],\qquad \tilde{W}_{c} := \left[ \begin{array}{cc} Q_{c} &~C_{c}^{\mathsf{H}} \\ C_{c} &~R_{c} \end{array} \right], $$

and

$$ \tilde{W}_{c}(X_{c}) := \left[ \begin{array}{cc} Q_{c} & C_{c}^{\mathsf{H}}\\ C_{c} & R_{c} \end{array} \right] - \left[\begin{array}{cc} A^{\mathsf{H}}_{c} & I \\ B^{\mathsf{H}}_{c} & 0 \end{array} \right] \left[\begin{array}{cc} 0 & X_{c} \\ X_{c} & 0 \end{array} \right] \left[\begin{array}{cc} A_{c} & B_{c} \\ I & 0 \end{array} \right]. $$

Note, that $\tilde {W}_{c}(X_{c})$ differs from *W*_*c*_(*X*_*c*_) as defined in (4) by a constant summand, i.e.,

$$ \tilde{W}_{c}(X_{c}) = W_{c}(X_{c}) + \left[\begin{array}{cc} Q_{c} & 0 \\ 0 & 0 \end{array}\right]. $$

Then we obtain a transformed discrete-time system by setting

26 $$ \begin{array}{@{}rcl@{}} A_{d} & :=& (-I + A_{c})^{-1}(I+A_{c})\\ B_{d} & :=& \sqrt{2}(-I + A_{c})^{-1}B_{c}\\ \tilde{W}_{d} & :=& \left[ \begin{array}{cc} Q_{d} & C_{d}^{\mathsf{H}}\\ C_{d} & R_{d} \end{array} \right]:= Z^{\mathsf{H}}_{c}\tilde{W}_{c}Z_{c}\\ \tilde{W}_{d}(X_{d})&:=& Z^{\mathsf{H}}_{c}\tilde{W}_{c}(X_{d})Z_{c}, \end{array} $$

where *Q*_*d*_, *C*_*d*_ and *R*_*d*_ are obtained from $\tilde {W}_{d}$ and we choose some *D*_*d*_ such that $R_{d}= D_{d} + D_{d}^{\mathsf H}$. This defines a mapping $\mathcal C$ with $\mathcal C (A_{c}, B_{c},C_{c}, R_{c}) = (A_{d}, B_{d}, C_{d}, R_{d})$. Note, that the transformation $\mathcal C$ can also be reversed.

Bilinear transformations preserve asymptotic stability, and they also relate the domains of the continuous-time and discrete-time linear matrix inequalities. To see this, we express the two LMIs as

$$ \begin{array}{@{}rcl@{}} \tilde{W}_{c}(X_{c}) &=& \left[\begin{array}{cc} Q_{c} & C_{c}^{\mathsf{H}}\\ C_{c} & R_{c} \end{array} \right] - \left[\begin{array}{cc} A^{\mathsf{H}}_{c} & I \\ B^{\mathsf{H}}_{c} & 0 \end{array} \right] \left[\begin{array}{cc} 0 & X_{c} \\ X_{c} & 0 \end{array} \right] \left[\begin{array}{cc} A_{c} & B_{c} \\ I & 0 \end{array} \right]\succeq0,\\ \tilde{W}_{d}(X_{d}) &=& \left[ \begin{array}{cc} Q_{d} & C_{d}^{\mathsf{H}} \\ C_{d} & R_{d} \end{array} \right] - \left[\begin{array}{cc} A^{\mathsf{H}}_{d} & I \\ B^{\mathsf{H}}_{d} & 0 \end{array} \right] \left[\begin{array}{cc} X_{d} & 0 \\ 0 & -X_{d} \end{array} \right] \left[\begin{array}{cc} A_{d} & B_{d} \\ I & 0 \end{array} \right]\succeq0, \end{array} $$

respectively. Since

$$ \left[\begin{array}{cc} 0 & X_{c} \\ X_{c} & 0 \end{array} \right] = \left[\begin{array}{cc} I & I \\ I & -I \end{array} \right] \left[\begin{array}{cc} \frac{X_{c}}{2} & 0 \\ 0 & -\frac{X_{c}}{2} \end{array} \right] \left[\begin{array}{cc} I & I \\ I & -I \end{array} \right], $$

we can also express $\tilde {W}_{c}(X_{c})$ as

$$ \tilde{W}_{c}(X_{c})=\left[ \begin{array}{cc} Q_{c} & C_{c}^{\mathsf{H}} \\ C_{c} & R_{c} \end{array} \right] - \left[\begin{array}{cc} A_{c}+I & B_{c} \\ A_{c}-I & B_{c} \end{array} \right]^{\mathsf{H}} \left[\begin{array}{cc} \frac{X_{c}}{2} & 0 \\ 0 & -\frac{X_{c}}{2} \end{array} \right] \left[\begin{array}{cc} A_{c}+I & B_{c} \\ A_{c}-I & B_{c} \end{array} \right]. $$

Applying the congruence transformation *Z*_*c*_ defined in (26), then

$$ Z^{\mathsf{H}}_{c}\tilde{W}_{c}(X_{c})Z_{c}= \left[ \begin{array}{cc} Q_{d} & C^{\mathsf{H}}_{d} \\ C_{d} & R_{d} \end{array} \right] - \left[\begin{array}{cc} A^{\mathsf{H}}_{d} & I \\ B^{\mathsf{H}}_{d} & 0 \end{array} \right] \left[\begin{array}{cc} X_{d} & 0 \\ 0 & -X_{d} \end{array} \right] \left[\begin{array}{cc} A_{d} & B_{d} \\ I & 0 \end{array} \right], $$

with *A*_*d*_, *B*_*d*_, *C*_*d*_, *R*_*d*_ and *Q*_*d*_ defined as in (26). For the transformation of the matrices *C*_*c*_, *R*_*c*_, and *Q*_*c*_ we obtain

27 $$ \left[\begin{array}{cc} Q_{d} & C_{d}^{\mathsf{H}}\\ C_{d} & R_{d} \end{array}\right] = Z_{c}^{\mathsf{H}} \left[\begin{array}{cc} Q_{c} & C_{c}^{\mathsf{H}}\\ C_{c} & R_{c} \end{array}\right] Z_{c}= Z_{c}^{\mathsf{H}} \left[\begin{array}{cc} \sqrt2 Q_{c} (I-A_{c})^{-1} & Q_{c} (I-A_{c})^{-1} B_{c} + C_{c}^{\mathsf{H}}\\ \sqrt2 C_{c} (I-A_{c})^{-1} & C_{c} (I-A_{c})^{-1}B_{c} + R_{c} \end{array}\right], $$

where the respective quantities are given by

$$ \begin{array}{@{}rcl@{}} Q_{d} &=& 2 (I-A_{c})^{-\mathsf{H}}Q_{c} (I-A_{c})^{-1},\\ C_{d}^{\mathsf H}&=& \sqrt2 (I-A_{c})^{-\mathsf{H}}Q_{c} (I-A_{c})^{-1} B_{c} + \sqrt2 (I-A_{c})^{-\mathsf{H}}C_{c}^{\mathsf{H}},\\ R_{d} &=& (I-A_{c})^{-1}B_{c} +B_{c}^{\mathsf{H}}(I-A_{c})^{-\mathsf{H}}Q_{c}(I-A_{c})^{-1} B_{c} + B_{c}^{\mathsf{H}}(I-A_{c})^{-\mathsf{H}}C_{c}^{\mathsf{H}} + R_{c}. \end{array} $$

This shows, that maximizing $\det W_{d}(X_{d})$ over *X*_*d*_ and maximizing $\det W_{c}(X_{c})$ over *X*_*c*_ is equivalent. In particular, this holds when *Q*_*c*_ = 0 which is equivalent to *Q*_*d*_ = 0. Thus, the respective continuous-time analytic center *X*_*a*,*c*_ and the discrete-time analytic center *X*_*a*,*d*_ coincide, i.e., *X*_*a*,*c*_ = *X*_*a*,*d*_.

It is well-known, that the bilinear transformation also preserves the solution of the algebraic Riccati equation as well as the domain of the linear matrix inequality $\tilde {W}_{c}(X_{c})\succeq 0$.

### B.2 Transformation of the Deflating Dubspaces

Following [7] we consider the pencils

$$ s\mathcal{E}_{c} - \mathcal{A}_{c} := \left[\begin{array}{ccc} 0     & -sI+A_{c} & B_{c}  \\ sI+ A_{c}^{\mathsf{H}}   & Q_{c} & C_{c}^{\mathsf{H}} \\ B_{c}^{\mathsf{H}}    & C_{c} & R_{c} \end{array} \right] $$

corresponding to the continuous-time case and

$$ z\mathcal{A}_{d}^{\mathsf{H}} - \mathcal{A}_{d} := \left[\begin{array}{ccc} 0 &~ zI-A_{d} &~ -B_{d}\\ zA_{d}^{\mathsf{H}}-I &~(z-1)Q_{d} &~(z-1) C_{d}^{\mathsf{H}} \\ zB_{d}^{\mathsf{H}} &~(z-1)C_{d} &~(z-1)R_{d} \end{array}\right] $$

corresponding to the discrete-time case, where $(A_{d}, B_{d}, C_{d}, R_{d})=\mathcal C (A_{c}, B_{c},C_{c}, R_{c})$, see (26). If *X*_*r*,*d*_ is a solution of *R**i**c**c*_*d*_(*X*_*r*,*d*_) = −*Q*_*d*_, then there is a deflating subspace of the form

$$ \begin{array}{@{}rcl@{}} &&\left[\begin{array}{ccc} 0 &~A_{d} - zI &~B_{d} \\ I -zA_{d}^{\mathsf{H}} &~(z-1)Q_{d} &~(z-1)C_{d}^{\mathsf{H}}\\ -zB_{d}^{\mathsf{H}} &~(z-1) C_{d} &~(z-1)R_{d} \end{array}\right] \left[\begin{array}{c} -X_{r,d}\left( I - A_{d} + B_{d}F_{r,d} \right)\\ I\\ -F_{r,d} \end{array}\right]\\ &&\qquad = \left[\begin{array}{c} I\\ (I-A_{d}^{\mathsf{H}})X_{r,d}\\ -B_{d}^{\mathsf{H}}X_{r,d} \end{array}\right] \left( A_{d} - B_{d}F_{r,d} - zI\right). \end{array} $$

Applying a generalized bilinear transformation to the pencil $s\mathcal {E}_{c} -\mathcal {A}_{c}$ gives

$$ \begin{array}{@{}rcl@{}} z \hat{\mathcal{A}}_{d} - \hat{\mathcal{A}}^{\mathsf{H}}_{d} &:=& z(\mathcal{E}_{c}-\mathcal{A}_{c}) - (-\mathcal{E}_{c}-\mathcal{A}_{c}) \\ &=& \left[\begin{array}{ccc} 0 &~z(A_{c}-I) - (I+A_{c}) &~zB_{c} - B_{c} \\ -z(I +A_{c}^{\mathsf{H}}) - (A_{c}^{\mathsf{H}} - I) &~(z-1)Q_{c} &~(z-1)C_{c}^{\mathsf{H}} \\ -zB_{c}^{\mathsf{H}} - B_{c}^{\mathsf{H}} &~(z-1) C_{c} &~(z-1)R_{c} \end{array}\right], \end{array} $$

and then performing a congruence transformation using *Z*_*c*_ from (25), we obtain the new pencil

$$ z\check{\mathcal{A}_{d}} - \check{\mathcal{A}_{d}}^{\mathsf{H}} := \left[\begin{array}{cc} \frac1{\sqrt2}I & 0 \\ 0 & Z_{c}^{\mathsf{H}} \end{array}\right] \left( z \hat{\mathcal{A}_{d}} - \hat{\mathcal{A}_{d}}^{\mathsf{H}}\right) \left[\begin{array}{cc} \frac1{\sqrt2} I & 0 \\ 0 & Z_{c} \end{array}\right] = \left[\begin{array}{ccc} 0 &~A_{d} - zI &~B_{d} \\ I -zA_{d}^{\mathsf{H}} &~(z-1)Q_{d} &~(z-1)C_{d}^{\mathsf{H}}\\ -zB_{d}^{\mathsf{H}} &~(z-1)C_{d} &~(z-1)R_{d} \end{array}\right]. $$

If, conversely, there is a continuous-time solution *X*_*r*,*c*_ of *R**i**c**c*_*c*_(*X*_*r*,*c*_) = −*Q*_*c*_, we have the deflating subspace

$$ \left[\begin{array}{ccc} 0 & -sI+A_{c} & B_{c} \\ sI+ A_{c}^{\mathsf{H}} & Q_{c} & C_{c}^{\mathsf{H}} \\ B_{c}^{\mathsf{H}} & C_{c} & R_{c} \end{array}\right] \left[\begin{array}{c} -X_{r,c}\\ I\\ -F_{r,c} \end{array}\right] = \left[\begin{array}{c} I\\ X_{r,c}\\ 0 \end{array}\right] \left( A_{c}- B_{c}F_{r,c} - sI\right). $$

Then, using the same transformation we obtain

$$ \begin{array}{@{}rcl@{}} &&\left( z\check{\mathcal{A}_{d}} - \check{\mathcal{A}_{d}}^{\mathsf{H}}\right) \left[\begin{array}{cc} \sqrt2 I & 0 \\ 0 & Z_{c}^{-1} \end{array}\right] \left[\begin{array}{c} -X_{r,c}\\ I\\ -F_{r,c} \end{array}\right] (I-A_{c}+B_{c}F_{r,c})^{-1}\\ && \qquad= \left[\begin{array}{cc} \frac1{\sqrt2}I & 0 \\ 0 & Z_{c}^{\mathsf{H}} \end{array}\right] \left[\begin{array}{c} I\\ X_{r,c}\\ 0 \end{array}\right] \big(z(-I + A_{c}- B_{c}F_{r,c}) - (I+ A_{c} - B_{c}F_{r,c})\big)(I-A_{c}+B_{c}F_{r,c})^{-1}, \end{array} $$

which is equivalent to

$$ \left( z\check{\mathcal{A}_{d}}- \check{\mathcal{A}_{d}}^{\mathsf{H}}\right) \left[\begin{array}{c} - X_{r,c} (I-A_{F_{r,d}})\\ I\\ -\sqrt2 F_{r,c}(I-A_{c}+B_{c}F_{r,c})^{-1} \end{array}\right] = \left[\begin{array}{c} I\\ (I-A_{d})^{\mathsf{H}}X_{r,c}\\ -B_{d}^{\mathsf{H}}X_{r,c} \end{array}\right] (A_{F_{r,d}} -zI), $$

where $A_{F_{r,d}}$ denotes the bilinear transform of the matrix $A_{F_{r,c}} = A_{c}-B_{c}F_{r,c}$. One can check that $A_{F_{r,d}}$ fulfills $A_{F_{r,d}} = A_{d} - B_{d}F_{r,d}$ with

28 $$ F_{r,d} = \sqrt2 F_{r,c}(I-A_{c}+B_{c}F_{r,c})^{-1}. $$

In summary, we have shown the following.

#### **Theorem 1**

Let *X*_*r*,*c*_ solve the algebraic Riccati equation *R**i**c**c*_*c*_(*X*_*r*,*c*_) = −*Q*_*c*_. Then, *X*_*r*,*c*_ also solves the discrete-time Riccati equation *R**i**c**c*_*d*_(*X*_*r*,*c*_) = −*Q*_*d*_, where the corresponding Riccati feedback *F*_*r*,*d*_ is given by (28).

Also, the corresponding closed-loop matrices $A_{F_{r,c}}$ and $A_{F_{r,d}}$ are related via the bilinear transformation.

For the analytic center though, we have seen in Section 3, that the corresponding closed-loop matrices $A_{F_{c}}$ and $A_{F_{d}}$ are not related via a bilinear transform, as all eigenvalues of $A_{F_{c}}$ lie on the imaginary axis, while all eigenvalues of $A_{F_{d}}$ lie *inside* the unit circle.

In the remaining part of this section, we give some more explanation for that difference. In particular, we rewrite the continuous-time solution of the analytic center into an appropriately formulated Riccati equation approach, and, after applying the bilinear transformation and enforcing the discrete-time feedback *F*_*r*,*d*_, compare this with its discrete counterpart.

Let us again denote the continuous-time analytic center by *X*_*a*,*c*_. Thus, it fulfills the relation *P*_*c*_(*X*_*a*,*c*_) = *R**i**c**c*_*c*_(*X*_*a*,*c*_) and hence solves *R**i**c**c*_*c*_(*X*_*a*,*c*_) = −*Q*_*c*_, where *Q*_*c*_ := −*P*_*c*_(*X*_*a*,*c*_). Consequently, using the bilinear transformation and since *X*_*a*,*c*_ = *X*_*a*,*d*_, also *R**i**c**c*_*d*_(*X*_*a*,*d*_) = −*Q*_*d*_ holds. Note, though, that the coefficients *C*_*d*_,*R*_*d*_ contained in *R**i**c**c*_*d*_(*X*) and *Q*_*d*_, explicitly depend on *Q*_*c*_. Thus, they do not represent the discrete-time system with *Q*_*c*_ = *Q*_*d*_ = 0 that is used for the computation of the analytic center.

Consequently, even though one could expect that also *P*_*d*_(*X*_*a*,*d*_) = −*Q*_*d*_, this turns out not to be true. Furthermore, let us compute the quantity $\tilde {P}_{d}$, by enforcing the feedback *F*_*r*,*d*_ given by (28) via the following relation (leaving out the explicit dependence on *X*_*a*,*d*_)

$$ \left[\begin{array}{cc} \tilde{P}_{d} &0 \\ 0 &R_{d} - B_{d}^{\mathsf{H}}X_{a,d}B_{d} \end{array}\right] = (Z_{P})^{\mathsf{H}} \left[\begin{array}{cc} P_{c} & 0 \\ 0 & R_{c} \end{array}\right] \underbrace{ \left[\begin{array}{cc} I & 0 \\ F_{r,c} & I \end{array}\right] \left[\begin{array}{cc} \sqrt{2}(I-A_{c})^{-1} &(I-A_{c})^{-1}B_{c}\\ 0 &I \end{array}\right] \left[\begin{array}{cc} I & 0 \\ -F_{r,d} & I \end{array}\right]}_{Z_{P}:=}, $$

where we form

$$ Z_{P} = \left[\begin{array}{cc} \sqrt2 (I - A_{c}+B_{c}F_{r,c})^{-1}& (I - A_{c})^{-1}B_{c}\\ 0 & I + F_{r,c}(I - A_{c})^{-1}B_{c} \end{array}\right], $$

and have used (28) and that $\sqrt 2 F_{r,c}(I - A_{c})^{-1} - F_{r,d} -F_{r,c}(I - A_{c})^{-1}B_{c}F_{r,d}=0$. We thus obtain that

$$ \tilde{P}_{d} = 2 (I - A_{c}+B_{c}F_{r,c})^{-\mathsf{H}}P_{c} (I - A_{c}+B_{c}F_{r,c})^{-1}, $$

which, by considering that *P*_*c*_ ≻ 0 and (27), only coincides with − *Q*_*d*_ if *F*_*r*,*c*_ = 0.

In particular, we have shown, that if we enforce a discrete feedback *F*_*r*,*d*_ as in (28), that keeps the eigenvalues of the closed-loop matrix $A_{F_{d}}$ at the analytic center on the unit circle, then $\tilde {P}_{d}\neq -Q_{d}$ and thus does not equal the discrete-time residual *P*_*d*_(*X*_*a*,*d*_). Indeed, as mentioned before, the eigenvalues of $A_{F_{d}}$ lie strictly inside the unit circle.
